# PDGF-BB enhances collagen gel contraction through a PI3K-PLCγ-PKC-cofilin pathway

**DOI:** 10.1038/s41598-017-08411-1

**Published:** 2017-08-21

**Authors:** Vahid Reyhani, Maria Tsioumpekou, Tijs van Wieringen, Lars Rask, Johan Lennartsson, Kristofer Rubin

**Affiliations:** 1 Department of Medical Biochemistry and Microbiology, Science for Life Laboratory, Uppsala University, BMC Box 582, SE-751 23 Uppsala, Sweden; 2 Ludwig Institute for Cancer Research, Science for Life Laboratory, Uppsala University, Box 595, SE-751 24 Uppsala, Sweden

## Abstract

Cell-mediated contraction of collagenous matrices is modulated by various growth factors and cytokines, such as platelet-derived growth factor-BB (PDGF-BB). Here we used a genetic cell model to delineate defined signaling pathways that enhance collagen gel contraction downstream of ligand-stimulated platelet-derived growth factor receptor-β (PDGF-Rβ). Our data show that PDGF BB-enhanced activations of phosphatidylinositol 3′-kinase (PI3K) and phospholipase Cγ (PLCγ) were necessary for PDGF-enhanced collagen gel contraction. Importantly, other defined signaling pathways down-stream of PDGF-Rβ were, however, dispensable. The decisive roles for PI3K and PLCγ were corroborated by experiments using selective inhibitors. Furthermore, we show that de-phosphorylation and thereby activation of cofilin that is important for the turnover of actin filaments, is depended on PI3K and PLCγ down-stream of PDGF-Rβ. Moreover, inhibition of protein kinase C (PKC) by GÖ6976 and bisindolylmaleimide-II abolished cofilin de-phosphorylation, as well as PDGF-enhanced contraction. In contrast, activation of the PKC protein family by 4β-phorbol 12-myristate 13-acetate (PMA) did not accelerate collagen gel contraction although it induced long-term cofilin de-phosphorylation, showing the need of a dynamic control of cofilin de-phosphorylation for PDGF-enhanced collagen gel contraction. Taken together, our data point to the involvement of a PI3K/PLCγ-PKC-cofilin pathway in both PDGF-enhanced cofilin de-phosphorylation and PDGF-enhanced collagen gel contraction.

## Introduction

Cell-mediated contraction of the interstitial extracellular matrix (ECM) controls the interstitial fluid content^1^. The cell-mediated matrix contraction is controlled by cytoplasmic signaling events that modulate the generation of the mechanoforces that cells apply on the ECM via integrins^2–7^. Cell-mediated collagen gel contraction *in vitro* can be used as a model to study this *in vivo* process^8–13^.

Platelet-derived growth factor-BB (PDGF-BB) stimulates cell-mediated collagen gel contraction^14^. Platelet-derived growth factor receptors (PDGF-R) are tyrosine kinase receptors consisting of receptor-α and receptor-β isoforms^15, 16^. Upon ligand binding, the PDGF-R undergoes dimerization, which leads to auto-phosphorylation of several tyrosine residues initiating signaling pathways. Among the 5 isoforms of PDGF, only PDGF-BB can bind both PDGF-Rα and PDGF-Rβ^15^. PDGF BB-stimulated signaling through PDGF-Rβ, but not PDGF-AA/PDGF-Rα, is involved in fluid homeostasis and regulation of the interstitial fluid pressure in normal rat dermis by regulating the cell-mediated tissue contraction^9, 13^. This effect is through signaling events that are not fully described.

Studies using mutational or inhibitory approaches have provided evidence for a crucial role of phosphatidylinositol 3′-kinase (PI3K) in PDGF-enhanced actin turnover and chemotaxis^17, 18^, cell growth^19, 20^, as well as for collagen gel contraction *in vitro* and normalization of anaphylaxis-induced lowered dermal interstitial fluid pressure *in vivo* ^12, 21^. The migratory response of cells to PDGF-BB is also dependent on PI3K^22–25^. Binding of p85, the regulatory subunit of PI3K, to phosphorylated tyrosine residues Y740 and/or Y751 of the PDGF-Rβ leads to activation of the catalytic subunit p110, which in turn phosphorylates phosphatidylinositol 4,5-bisphosphate (PIP_2_) to phosphatidylinositol 3,4,5-triphosphate (PIP_3_). Upon PDGF-Rβ activation, phospholipase Cγ (PLCγ) binds to phosphorylated Y1009 and Y1021 residues leading to its phosphorylation and activation^26^. Furthermore, available data suggest that, in response to growth factors, interaction of PIP_3_ with the PH-domain in PLCγ is required for membrane targeting and full activation of PLCγ^27, 28^. PLCγ induces hydrolysis of membrane-associated PIP_2_ resulting in the formation of diacylglycerol (DAG) and inositol 1,4,5-triphosphate (IP_3_). These products activate protein kinase C (PKC) and increase intracellular Ca^2+^ ^27, 28^. Furthermore, PI3K-generated PIP3 is involved in dissociation of activated PLCγ from PDGF-R and its recruitment to the cell membrane where it hydrolyses PIP2^29^. Similar to PI3K, PLCγ is also involved in chemotactic signaling events, suggesting that both are operational during chemotaxis^30, 31^. For example, the PI3K-induced activation of PLCγ is important during EGF-mediated chemotaxis^32^. In line with this, a PDGF-Rβ mutation that leads to an overactive PLCγ resulted in a PI3K-independent hyperchemotaxis^33, 34^. These observations suggest a dynamic interplay between PI3K and PLCγ signaling during cell-mediated matrix contraction.

Cofilin is a 19 kD actin binding and severing protein that through mediating actin turnover participates in cell motility (reviewed in^35^). Phosphorylation of cofilin by the family of LIM kinases (LIMK) leads to its inactivation^36–40^, while de-phosphorylation of phospho-cofilin (p-cofilin) by the family of Slingshot (SSH) phosphatases^38, 41, 42^ or chronophin^43^ activates it. Membrane-bound cofilin (not phosphorylated) is inactive when bound to PIP_2_ and can be locally activated through PLCγ-induced hydrolysis of PIP_2_ leading to the localized release of active cofilin^44^.

Previously we have shown that PDGF-BB induces de-phosphorylation and thereby activation of cofilin^45^. According to published studies, the cofilin activity in turnover of the actin cytoskeleton during cellular motility responses is fine-tuned through a dynamic balance between (*i*) p-cofilin de-phosphorylation by SSH, (*ii*) cofilin phosphorylation by LIMK, and (*iii*) PLCγ-induced release of membrane-bound cofilin (reviewed in ref. 35). For example, it has been shown that in human aortic smooth muscle cells, PDGF-BB dually regulates migration via activation of both LIMK and the SSH1L^46^. Moreover, knock-down of cofilin in myofibroblasts abolished collagen gel contraction by these cells^47^. The PI3K-PAK1-LIMK-cofilin pathway is involved in regulation of the PDGF-enhanced contraction of collagen gels^48^. Selective activators of PKC or cellular Ca^2+^ are shown to trigger de-phosphorylation of cofilin in neutrophils, whereas inhibitors of PKC blocked cofilin de-phosphorylation despite the presence of the PKC activator 4β-phorbol 12-myristate 13-acetate (PMA)^49^. Also, PAK4 is an important dual regulator of cofilin. It phosphorylates both SSH and LIMK^50^, providing another evidence for the existence of a dynamic balance between positive and negative regulators in cofilin activation.

Here we investigated the signaling events downstream of PDGF-Rβ during PDGF-enhanced collagen gel contraction, with the main focus on the signaling pathway through which PDGF-Rβ induces cofilin de-phosphorylation. We show that the PI3K/PLCγ-PKC pathway links the activated PDGF-Rβ to cofilin de-phosphorylation and this chain of signaling events is involved in PDGF-enhanced collagen gel contraction.

## Results

### PDGF-BB enhances collagen gel contraction in a PI3K and PLCγ dependent fashion

**Figure 1:**
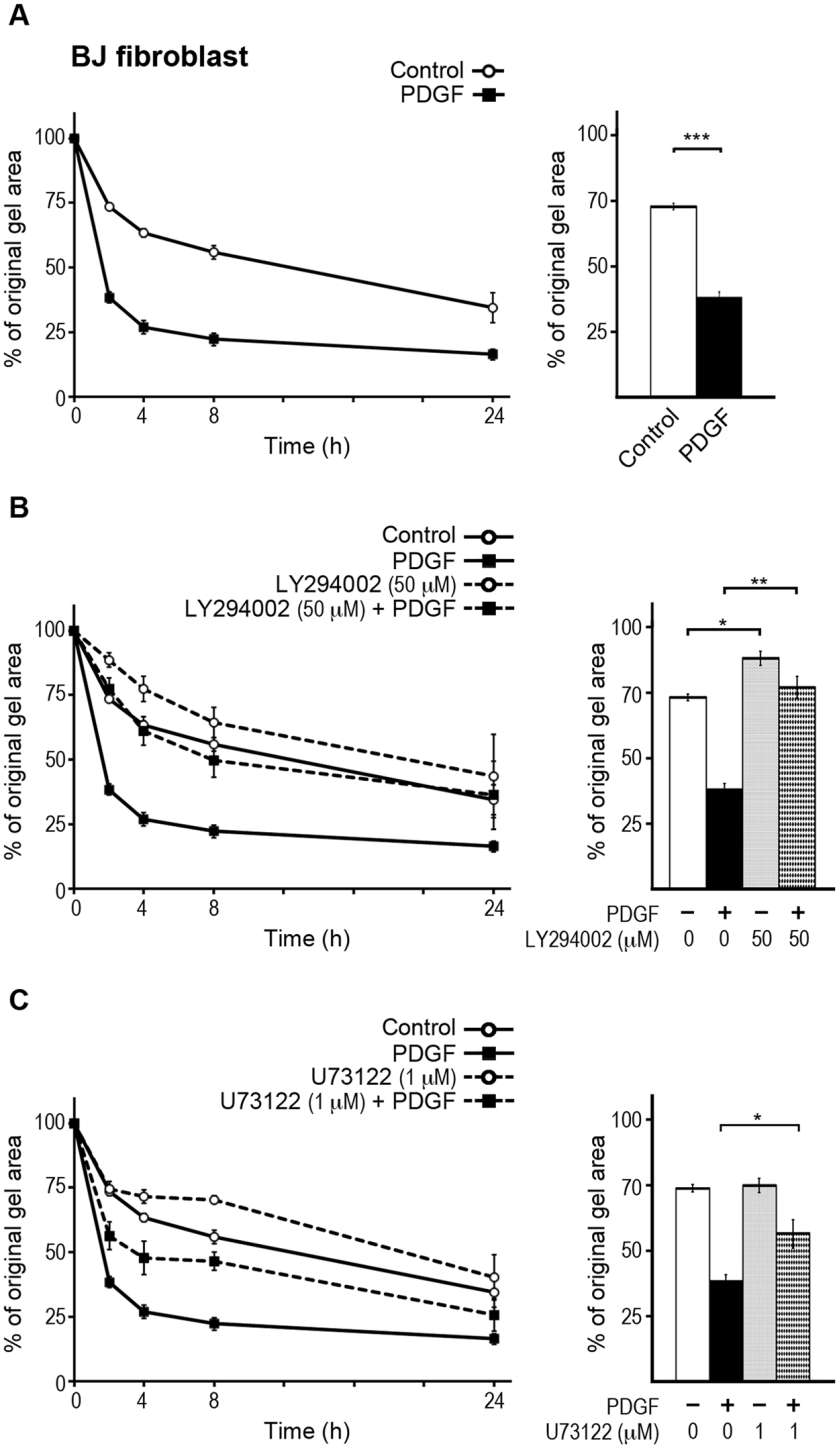
PI3K or PLCγ inhibition reduces PDGF-enhanced collagen gel contraction. (**A**) Collagen gel contraction by BJ fibroblasts were studied in presence or absence of 20 ng/mL PDGF-BB. The line graph (Left) presents the dynamic of contraction during 24 h, and bar chart (Right) presents the contraction after 2 h to illustrate the early effect of PDGF-BB on contraction. (**B**,**C**) BJ fibroblast cells were treated with the PI3K inhibitor LY294002 (**B**) or the PLCγ inhibitor U73122 (**C**) and contraction proceeded in presence or absence of 20 ng/mL PDGF-BB. In all panels, values represent average of at least four independent experiments. (*) indicates p < 0.05, (**) indicates p < 0.01, and (***) indicates p < 0.001. Error bars are SEM.

BJ fibroblasts contracted plain CN-I gels in the absence of any exogenous stimuli but the addition of 20 ng/mL PDGF-BB enhanced the contraction (Fig. 1A), in line with previously published studies^14^. Addition of the PI3K inhibitor LY294002 at 50 μM completely inhibited PDGF-enhanced contraction of collagen gels mediated by BJ fibroblasts, and partially reduced contraction occurred in the absence of PDGF-BB (control condition) (Fig. 1B). Moreover, treatment of BJ fibroblasts with 1 μM U73122 reduced the PDGF-enhanced contraction while no inhibitory effect on control contraction during the first 2 h was observed (Fig. 1C). These findings were in line with previously published studies describing the involvement of PI3K and PLCγ in various cell motility responses^21, 27, 32–34^.

### A combined action of PI3K and PLCγ mediates PDGF-enhanced collagen gel contraction

**Figure 2:**
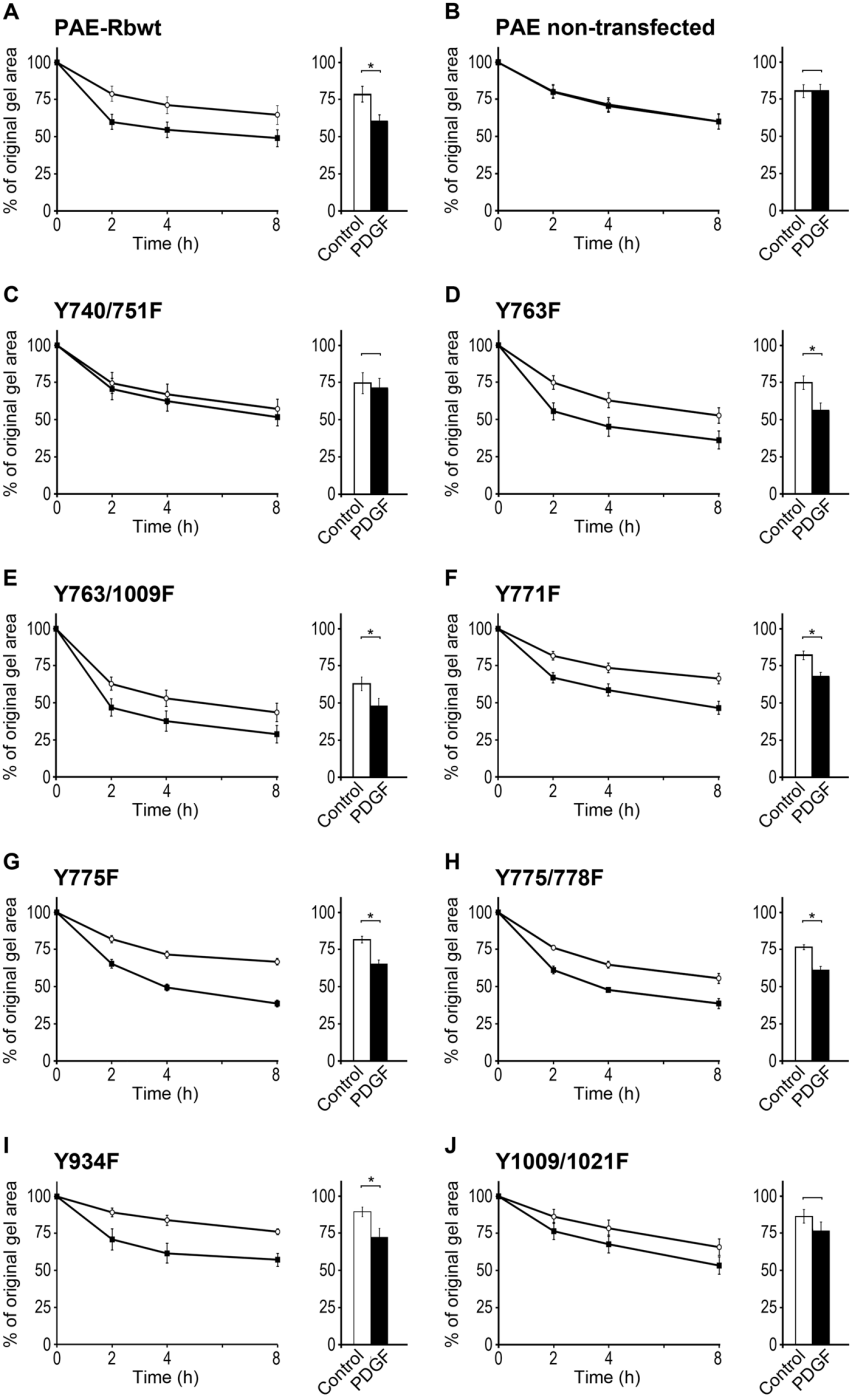
PI3K and PLCγ are both required for PDGF-enhanced contraction. (**A**) PAE-Rβwt, (**B**) non-transfected PAE cells that lack PDGF-Rβ, and (**C**–**J**) eight PAE-Rβ mutants (listed in Table 1), were used in collagen gel contraction assay. Among the investigated mutant cells, only the PAE-Rβ Y740/751F and Y1009/1021F, the mutants that were unable to activate PI3K and PLCγ respectively, were unable to respond to PDGF stimulation by an enhanced contraction. PDGF-stimulation had no effect on non-transfected PAE cells, lacking PDGF receptors. In contrast, the PAE-Rβwt (panel A) and the other six PAE-Rβ mutants (panels D–I) showed enhanced contraction in response to PDGF-BB. In all panels, the graph to the left presents the time-course of contraction up to 8 h and the bar chart to the right side presents the contraction after 2 h. Values are averages of a minimum of four independent experiments, each performed in triplicate. Error bars are SEM. (*) refers to p < 0.05.

The data presented in Fig. 1 that are based on selective inhibitors suggest that active PI3K and PLCγ are necessary for PDGF-enhanced contraction. To investigate whether only PI3K and PLCγ pathways are essential for PDGF-enhanced contraction or additional downstream PDGF-Rβ signaling cascades are involved, we took advantage of a unique *in vitro* cell model (PAE cell model), which allowed us to screen the effect of several individual signaling cascades downstream of PDGF-Rβ during PDGF-enhanced collagen gel contraction. The PAE cells, which lack endogenous PDGF-R, were transfected either with wild type human PDGF-Rβ or with receptors mutated at different specific tyrosine sites (as specified in Table 1). In each of these mutants, the PDGF-Rβ is impaired in the initiation of specific downstream signaling cascades, which allowed us to identify the crucial signaling cascades during PDGF-enhanced contraction. Contraction mediated by the PAE cells was studied in the presence or absence of PDGF-BB (Fig. 2). PAE cells expressing PDGF-Rβ carrying Y740/751F, that do not activate PI3K, or Y1009/1021F, that do not activate PLCγ, mutations were refractory with regard to PDGF BB-enhanced collagen gel contraction (Fig. 2C and J). In contrast, other PAE mutants and PAE-Rβwt responded to PDGF-BB by increased contraction compared to the control condition (Fig. 2). The investigated PAE cells with mutated PDGF-Rβ expressed similar levels of PDGF-Rβ mRNA and protein (data not shown). As a negative control, PAE non-transfected (PAE-NT) cells were used, which are the original PAE cells and lack endogenous expression of any isoform of PDGF-R. These data imply that both the PI3K and the PLCγ activation sites of PDGF-Rβ are essential for the PDGF-enhanced collagen gel contraction, whereas the other investigated PDGF-Rβ-elicited signal pathways were dispensable.

**Table 1:** List of PAE-Rβ mutants.

PAE-Rβ mutants	Target protein(s)
Y740/751F	PI3K and NcK
Y763F	SHP2
Y763/1009F	SHP2
Y771F	Ras and GAP
Y775F	Grb2 and Stat
Y775/778F	Grb2 and Stat
Y934F	
Y1009/1021F	PLCγ and SHP2

List of PAE-Rβ mutants and the signaling protein(s) that their mutated PDGF-Rβ receptor cannot interact with upon receptor activation.

### Cofilin plays a general role in collagen gel contraction

**Figure 3:**
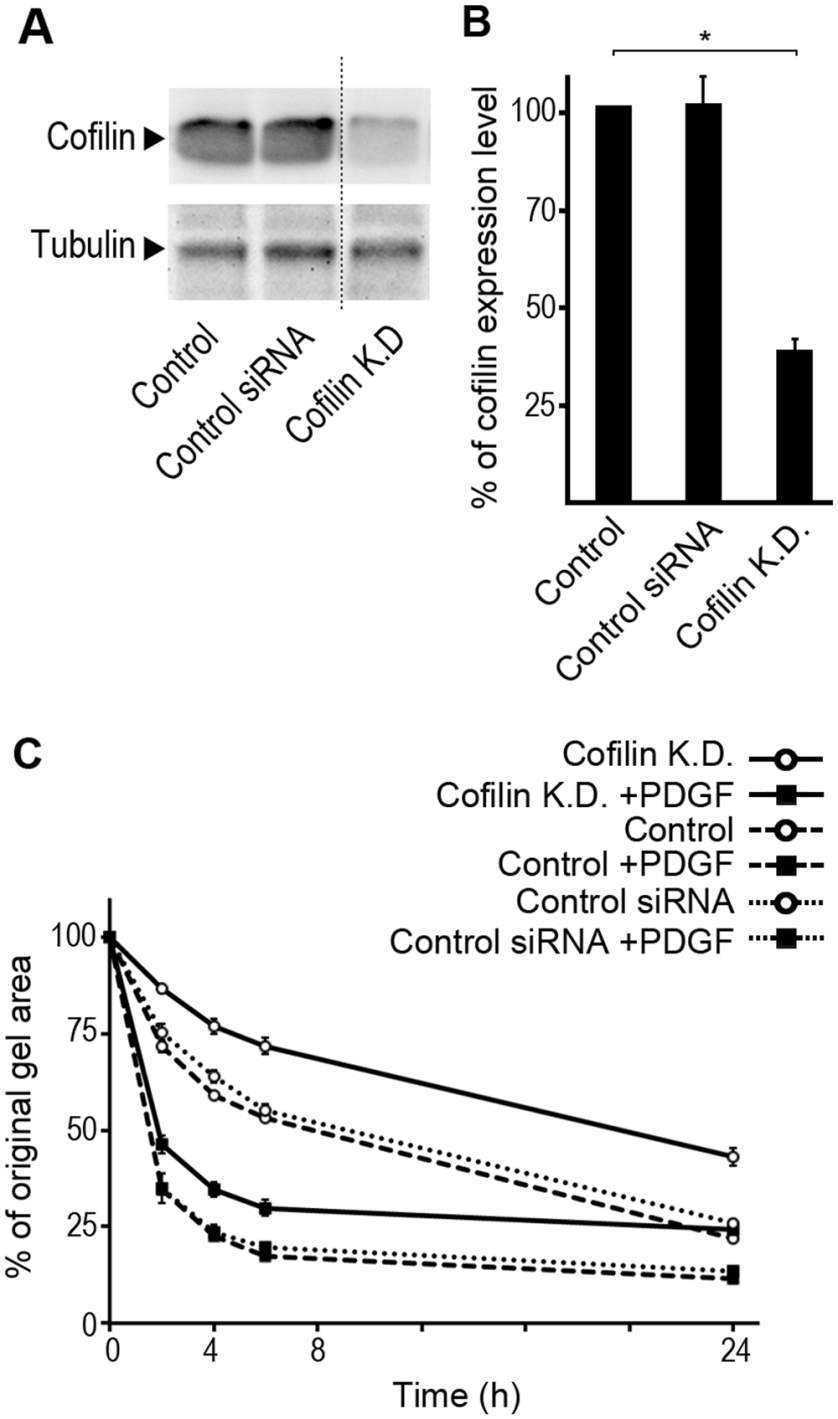
Knockdown of cofilin inhibits cell-mediated collagen gel contraction, both in presence or absence of PDGF-BB. (**A**,**B**) Detected and quantified protein level of cofilin (72 h after transfection) in BJ fibroblasts without any treatment (control), after transfection with siRNA against cofilin (cofilin K.D.), or treatment with control siRNA. Tubulin protein level was also detected as an internal control. (**C**) 72 h after transfection of BJ fibroblasts with cofilin siRNA, the ability of the cells to contract gels in presence or absence of PDGF-BB was investigated. In all panels the presented data is from at least three independent experiments each performed in triplicate. Error bars are SEM and (***) refers to p < 0.001. The hashed line in panel A shows where the blot was cut to take away other transfection conditions (day 1, 2, and 4) from the same blot. The full-length blot is presented in the supplementary data.

Actin turnover is one of the essential events required for PDGF BB-stimulated cell migration and chemotaxis^24, 25, 45^. To investigate whether cofilin activity is involved in cell-mediated collagen gel contraction, BJ fibroblasts were transfected with siRNA directed against cofilin. Transfection of cells for 72 h resulted in maximal reduction of the cofilin at the protein level (Fig. 3A,B), while treatment with control siRNA had no significant effect on cofilin protein level (Fig. 3A,B). BJ fibroblasts with reduced cofilin level (cofilin K.D.) had a reduced ability to contract collagen gels in the presence or absence of PDGF-BB compared to control cells (Fig. 3C). Similar effects were observed in PAE cells that were transfected with siRNA directed against cofilin (see Supplementary Fig. S1).

### PI3K and PLCγ mediate PDGF-induced cofilin de-phosphorylation

**Figure 4:**
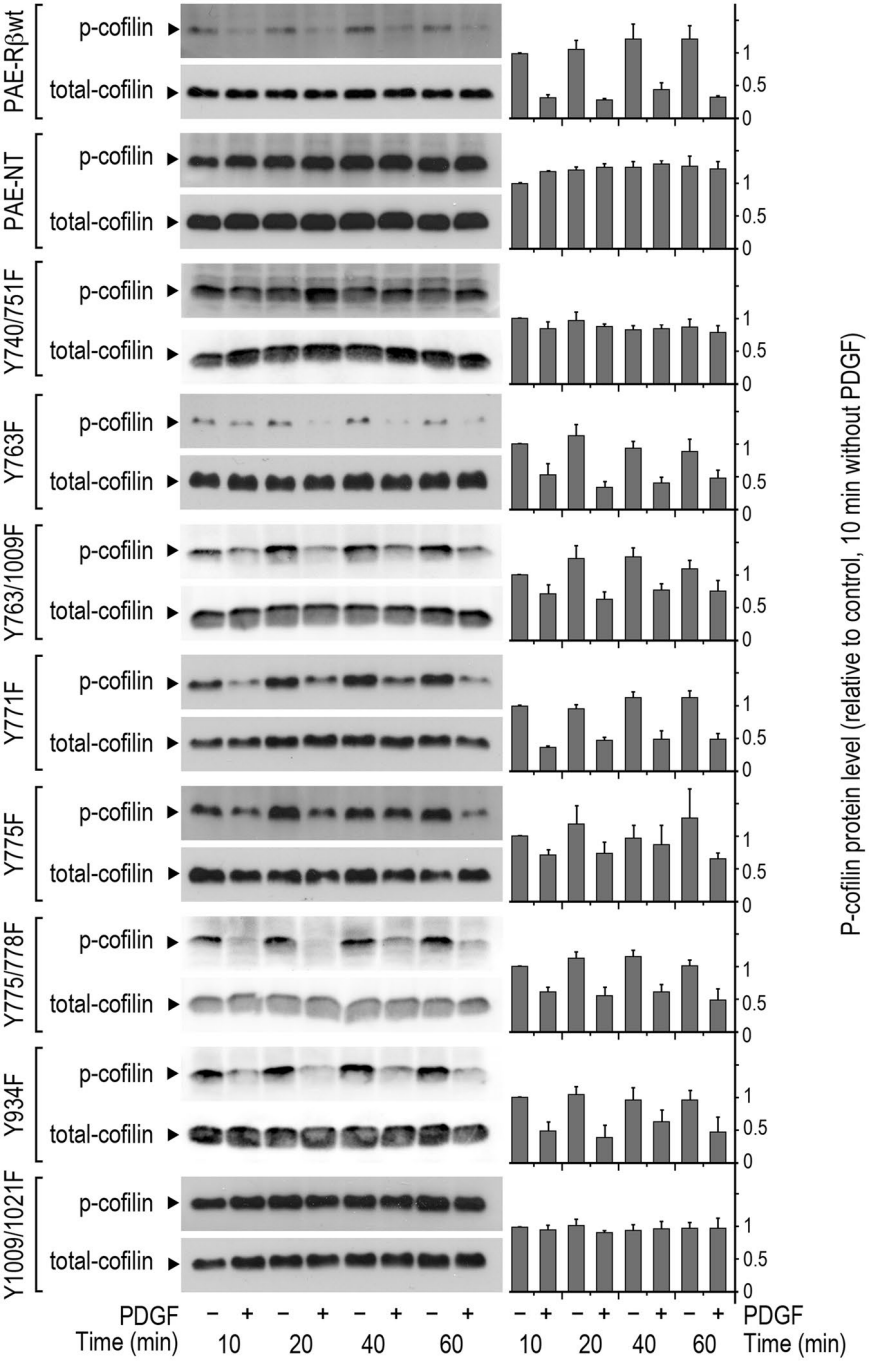
PI3K and PLCγ are both needed for PDGF-induced cofilin dephosphorylation. The levels of p-cofilin in PAE-Rβwt, non-transfected PAE cells (PAE-NT), and eight PAE-Rβ mutants (as listed in Table 1), after 10, 20, 40, and 60 min of stimulation with PDGF-BB (20 ng/mL). As an internal control, the total protein level of cofilin (phosphorylated and de-phosphorylated forms) was detected using the pan-cofilin antibody (total-cofilin). Representative blots of at least three independent experiments for each cell line are shown. The quantified data are averaged from at least three independent experiments. The values are normalized to respective levels of total-cofilin level, and are relative to the control condition (10 min without PDGF).

**Figure 5:**
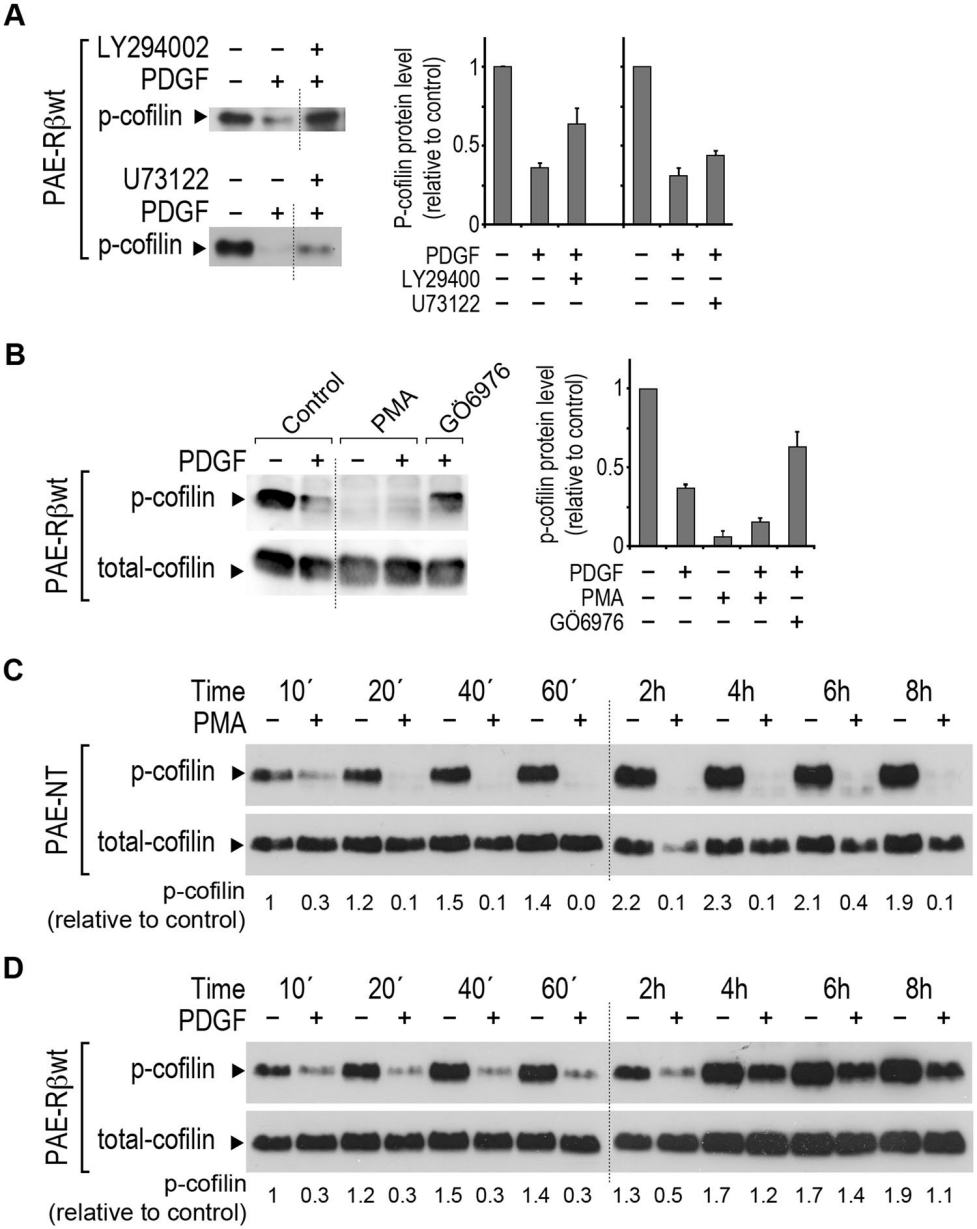
Inhibition of PI3K, PLCγ, and PKC inhibits PDGF-induced cofilin dephosphorylation. (**A**) Representative immunoblot of p-cofilin protein level in PAE-Rβwt cells after pre-treatment for 1 h with LY294002 (50 μM) or U73122 (10 μM), in the presence or absence of PDGF-BB (15 min stimulation). The quantified values are averaged from three independent experiments. (**B**) The effect on p-cofilin protein level by pre-treatment of PAE-Rβwt cells with the PKC inhibitor GÖ6976 (1 μM) 15 min after stimulation with PDGF-BB (20 ng/mL). The p-cofilin protein level was also quantified 15 min after stimulation of PAE-Rβwt cells with PMA in the presence or absence of PDGF-BB. The total protein level of cofilin (total-cofilin) was quantified as an internal control. The quantified values are normalized to the total-cofilin protein level, and relative to the control condition. (**C**) The protein level of p-cofilin and total-cofilin during 8 h stimulation of non-transfected PAE cells (PAE-NT) with PMA. (**D**) The protein level of p-cofilin and total-cofilin during 8 h of stimulation of PAE-Rβwt cells with PDGF-BB. In all panels shown data are representatives of at least three individual experiments. In panels (C and D) the quantified values are calculated as the ratio of p-cofilin band to total cofilin band and are presented below the corresponding bands. The hashed line in panels (A and B) show where the blot was cut to take away other tested conditions from the same blot. The hashed line in panels (C and D) show where two separate blots were placed next to each other to present the entire set of samples taken during the 8 h experiment together. The full-length copies of all the blots used in this figure are presented in supplementary information.

PDGF-BB induces de-phosphorylation and hence activation of cofilin^45^. Based on which, we investigated PDGF-induced cofilin de-phosphorylation to delineate signaling pathway(s) downstream of PDGF-Rβ that links the receptor activation to actin turnover and cell-mediated contraction. Again we took advantage of the PAE cell model. The levels of p-cofilin were quantified in different PAE mutants after stimulation with PDGF-BB (Fig. 4). PDGF-BB stimulation resulted in a rapid (within 10 min) decrease in p-cofilin levels in PAE-Rβwt, a decrease that was sustained for at least 60 min. PAE cells transfected with the PDGF-Rβ mutants Y740/751F and Y1009/1021F were unable to de-phosphorylate p-cofilin in response to PDGF-BB stimulation, in contrast to the PAE cells carrying other PDGF-Rβ mutations (Fig. 4). As an internal control, the total protein level of cofilin was quantified at the indicated time points (Fig. 4). To assure that the total protein level of cofilin can be used as an internal control, tubulin protein level was also quantified at the investigated time points after PDGF-BB stimulation, both in BJ fibroblasts and in PAE-Rβ cells (see Supplementary Fig. S2). Furthermore, PDGF-BB stimulation had no effect on protein levels of the PI3K and PLCγ (see Supplementary Fig. S2). These findings were in good agreement with the effects of treatment of PAE-Rβwt cells with the PI3K inhibitor, LY294002 (Fig. 5A), and the PLCγ inhibitor U73122 (Fig. 5A). Treatment of cells with LY294002 and wortmannin (data not shown) abolished the PDGF-induced cofilin de-phosphorylation (Fig. 5A). Treatment with U73122 also inhibited PDGF BB-stimulated de-phosphorylation of p-cofilin albeit less effectively than LY294002 (Fig. 5A).

Thus, PI3K and PLCγ function in concert both during PDGF-induced de-phosphorylation of p-cofilin and PDGF-enhanced collagen gel contraction (Fig. 2). Furthermore, the data suggest that PDGF BB-enhanced enhancement of collagen gel contraction involves de-phosphorylation of p-cofilin.

### PKC mediates PDGF-induced p-cofilin de-phosphorylation

Taken together, a collaborative function of PI3K and PLCγ is required for PDGF-BB induced cofilin de-phosphorylation, therefore a potential link between PI3K-PLCγ activity and cofilin de-phosphorylation was studied. Since members of the PKC protein family are downstream of the PLCγ-PIP_2_-DAG cascade, their involvement in cofilin de-phosphorylation was investigated. Treatment of the cells with the PKC inhibitors GÖ6976 (Fig. 5B) and bisindolylmaleimide-II (data not shown) reduced PDGF-induced cofilin de-phosphorylation compared to the control. Moreover, the PKC protein family activator 4β-phorbol 12-myristate 13-acetate (PMA) induced cofilin de-phosphorylation in the absence of PDGF-BB (Fig. 5B). Furthermore, stimulation of PAE-NT cells, which lack PDGF-R, with PMA (50 nM) resulted in cofilin de-phosphorylation (Fig. 5C), in analogy to the effect of PDGF-BB in PAE-Rβwt cells (Fig. 5D). However, PMA-induced cofilin de-phosphorylation was not reversible for up to 8 h (Fig. 5C), in contrast to the effect of PDGF-BB stimulation that was transient and the p-cofilin level was back to normal after 2 h (Fig. 5D).

### PKC mediates PDGF-enhanced collagen gel contraction

**Figure 6:**
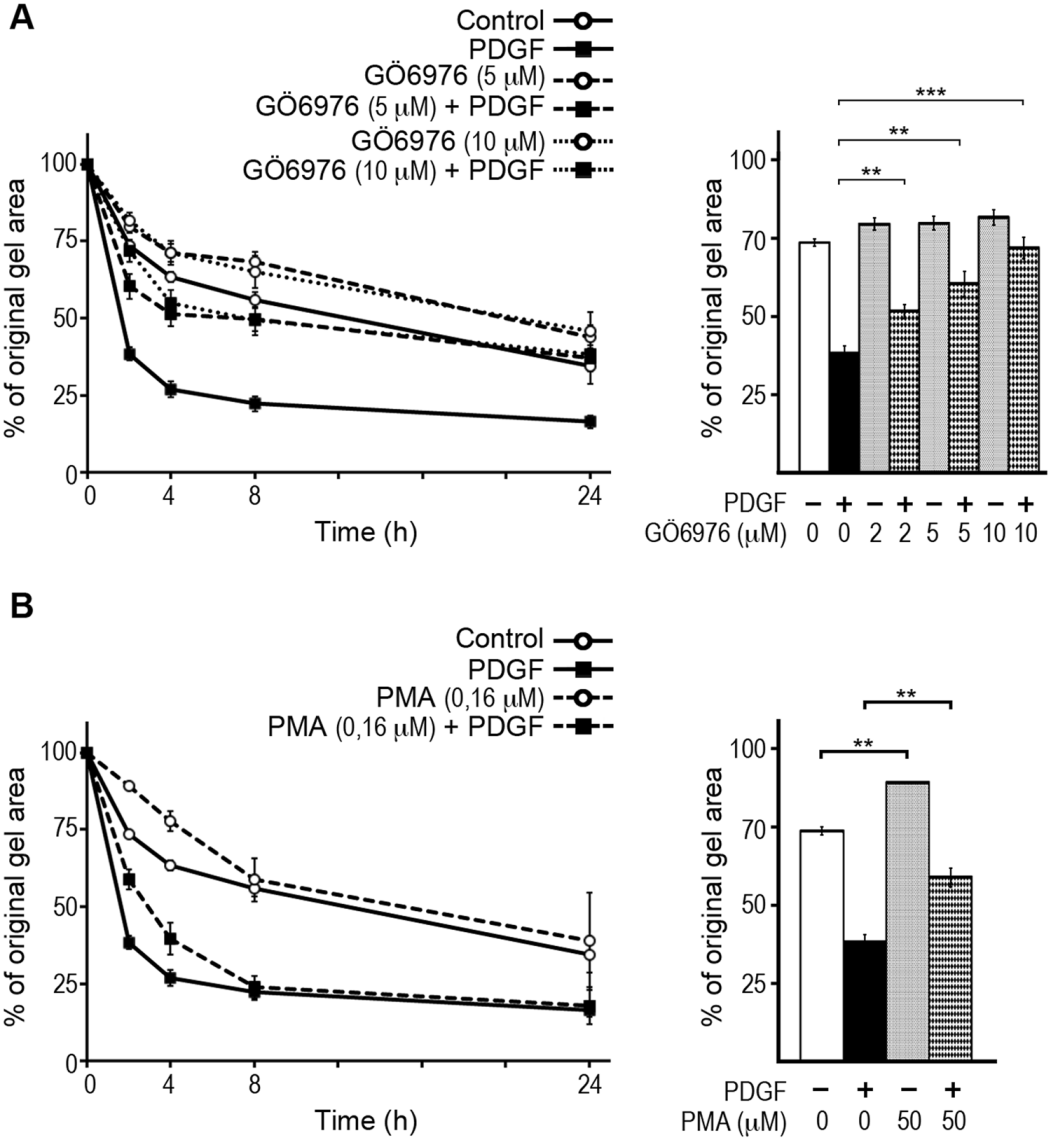
Inhibition of PKC inhibits PDGF-enhanced contraction of collagen gels. BJ fibroblast cells were treated with the PKC inhibitor GÖ6976 (**A**), or the PKC activator PMA (**B**) and used in collagen gel contraction in presence and absence of 20 ng/mL PDGF-BB. In both panels, the graph to the left presents the course of contraction during 24 h, while the bar chart to the right side presents the contraction after 2 h for statistical analysis. In both panels shown data are averaged from three individual experiments. (**) refers to p < 0.01 and (***) refers to p < 0.001. Error bars are SEM.

Addition of the PKC inhibitor GÖ6976 inhibited PDGF-enhanced contraction in a dose dependent manner (Fig. 6A, right panel). Treatment with GÖ6976 at 5 and 10 μM almost completely inhibited PDGF-enhanced contraction while it partially reduced the control contraction (Fig. 6A). Additionally, we investigated the potential impact of PMA treatment on collagen gel contraction by BJ fibroblasts. PMA not only failed to enhance the contraction despite inducing cofilin de-phosphorylation, but also reduced contraction in both the control and the PDGF-enhanced conditions during the first 8 h of contraction (Fig. 6B). The latter finding can be explained by the long-term effect of PMA on cofilin de-phosphorylation/activation (Fig. 5C), where an unbalanced long-term activation of cofilin (by de-phosphorylation) is not in favor of PDGF-enhanced collagen gel contraction.

## Discussion

Based on our comparative study between unique mutants of PDGF-Rβ, for the first time we show that only the phospho-tyrosine residues of PDGF-Rβ that activate PI3K and PLCγ initiate the signaling events required for PDGF-enhanced contraction as well as PDGF-induced p-cofilin dephosphorylation. Although the importance of PI3K or PLCγ during various cellular motility responses has been extensively discussed, further investigations on the signaling events and cross talks downstream of PI3K and PLCγ were, however, warranted. For example, the essential role of PI3K during actin turnover^22, 25^, migration^23, 24^, matrix contraction^21^, and regulation of interstitial fluid pressure^12^, as well as the importance of PLCγ in migration^32^ and chemotaxis^31^ has been described. In this study we provide evidence for an essential role of the collaborative function of PI3K and PLCγ during PDGF-enhanced ECM contraction. Our observations also provide a mediatory role for PKC in this signaling pathway, which links activated PDGF-Rβ to actin turnover through activating p-cofilin, as well as to gel contraction.

PI3K and PLCγ have an additive effect on cofilin activation, through release of membrane-bound cofilin as a localized activation at the cell margins and also through de-phosphorylation of cytoplasmic p-cofilin. Hence, depending on the method of cofilin activation, localized and/or overall turnover of actin can be triggered in favor of cellular motility responses downstream of PDGF-Rβ. In line with previous reports^48^, knock-down of cofilin with siRNA resulted in abrogation of contractile potency of BJ fibroblasts. This demonstrates that effective collagen gel contraction depends on the presence of cofilin. As shown earlier, in addition to receptor-mediated phosphorylation of PLCγ, PI3K is required for complete activation of PLCγ^27, 28^. In line with this note, our data suggest that PLCγ is downstream of both PDGF-Rβ and PI3K, and upstream of cofilin de-phosphorylation during PDGF-induced cofilin de-phosphorylation and gel contraction. Activated PLCγ hydrolyses PIP_2_, which in turn leads to release of active cofilin^44^. Our data on PDGF-induced cofilin de-phosphorylation suggest the PI3K-PLCγ-cofilin cascade as a secondary pathway downstream of PDGF-Rβ for cofilin activation by de-phosphorylation of p-cofilin.

PLCγ-hydrolysis of PIP_2_ activates classical members of the PKC protein family^51^. Strikingly, treatment of cells with the PKC inhibitors GÖ6976 and bisindolylmaleimide-II that inhibit the classical members of PKC protein family, led to reduced PDGF-induced de-phosphorylation of p-cofilin, as well as a reduced PDGF-enhanced contraction. In line with these observations, stimulation of cells with PMA, a PKC family protein activator, reduced p-cofilin levels independently of PDGF-BB stimulation. In contrast to PDGF-induced cofilin de-phosphorylation, PMA completely and irreversibly de-phosphorylated cofilin up to 8 hours. This may explain the observed partial inhibitory effect of PMA on contraction during the first 8 hours since stimulation of contraction, similar to other cell motility responses, requires a dynamic balance in actin turnover, which in turn is driven by fine-tuning of activation of actin-binding proteins such as cofilin. In other words, for cells to be able to complete a cellular motility response, activated cofilin has to be inactivated through phosphorylation by LIMK or via binding to PIP2 in the membrane. It has been previously shown that PDGF-Rβ induces LIMK activation^46, 48^, that together with our present findings point to the need of a dynamic balance between cofilin activation and de-activation downstream of the PDGF-Rβ, to enable PDGF-enhanced contraction. Lack of such a dynamic balance after stimulation of cells with PMA can explain the irreversibility of cofilin de-phosphorylation, as well as partial inhibition of contraction up to 8 hours. Together, these data suggest that PKC activation is downstream of PI3K and PLCγ and mediates cofilin de-phosphorylation and enhancement of contraction upon PDGF-Rβ activation. Suggestively, this pathway targets SSH and/or chronophin, which in turn activate cofilin via de-phosphorylation. Alternatively, PKC activation results in p-cofilin de-phosphorylation through inhibition of LIMK. However, LIMK is shown to be activated by PDGF-BB^46^, hence the effect of PKC on one of the SSH and/or chronophin seems more likely. According to Wang Y. *et al*. calcineurin, a Ca^2+^ dependent phosphatase, activates SSH which in turn de-phosphorylate p-cofilin^52^. This pathway potentially offers another mode of cofilin dephosphorylation upon PDGF-Rβ activation, since PLCγ activation leads to increase of cytosolic Ca^2+^ via IP_3_.

**Figure 7:**
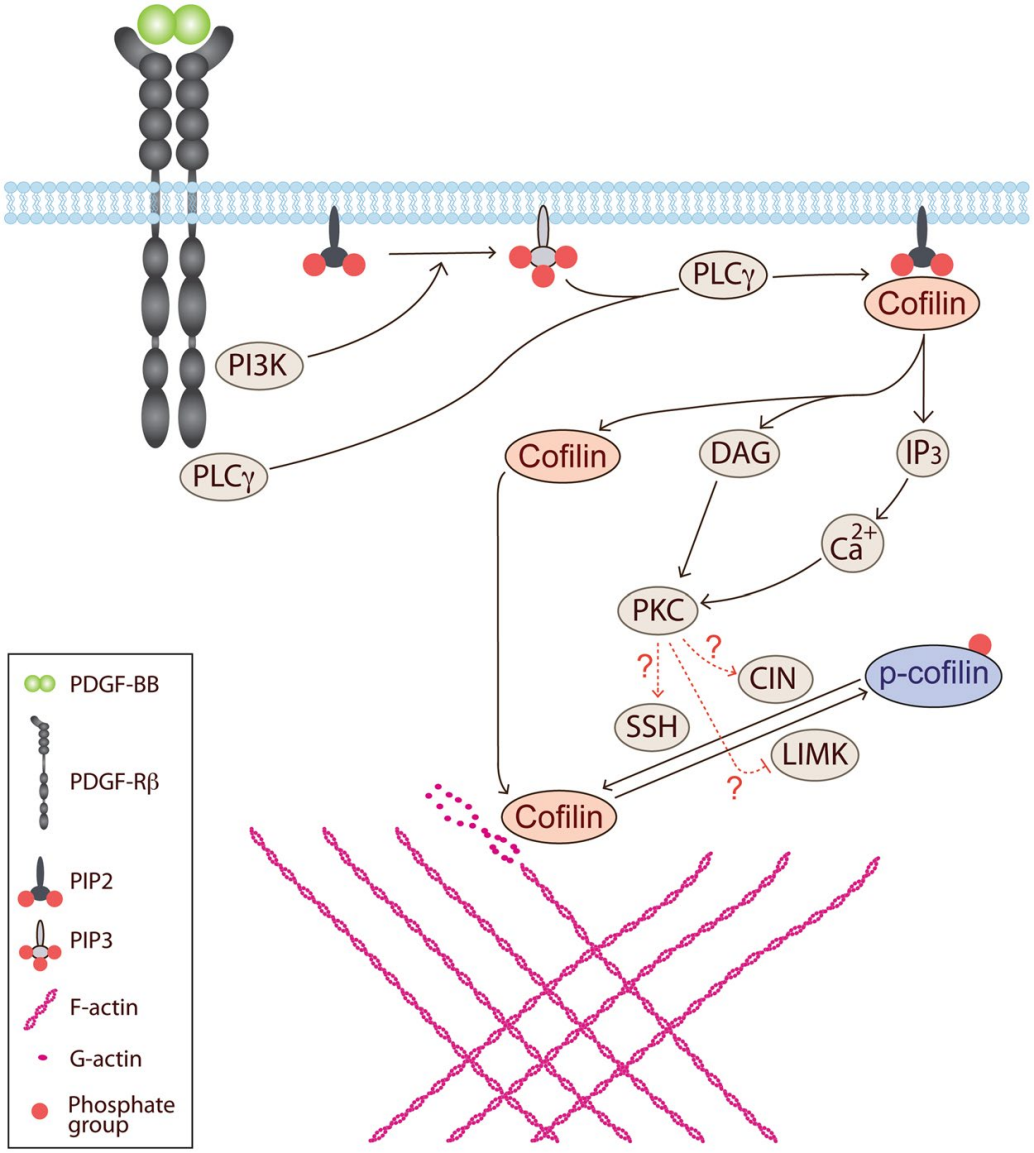
Proposed signaling events involved in PDGF-enhanced matrix contraction. Based on our findings, we propose that upon PDGF-Rβ activation, activation of PKC downstream of PI3K and PLCγ mediates PDGF-induced cofilin dephosphorylation during PDGF-enhanced collagen gel contraction. These findings point to activation of slingshot (SSH) and/or chronophin (CIN) phosphatases downstream of PKC, which requires further investigations.

In summary, we provide evidence for an essential role for PKC during the contraction of collagenous matrices. We also show that the paired function of PI3K and PLCγ downstream of PDGF-Rβ leads to PKC-dependent cofilin activation, which in turn triggers the actin turnover that is crucial for matrix contraction. Our data suggest that the PI3K-PLCγ-PKC-cofilin pathway is one of the crucial signaling events involved in PDGF-enhanced matrix contraction (Fig. 7).

## Materials and Methods

### Cells

BJhTERT immortalized human foreskin fibroblasts (BJ fibroblasts) were purchased from ATCC (CRL-4001). PAE cells that lack endogenous PDGF α- and β-receptors^53^ transfected with either wild-type human PDGF-Rβ (PAE-Rβwt) or the receptor variants bearing single or double tyrosine to phenylalanine point mutations^54^ were donated by Dr. Carl-Henrik Heldin (Ludwig Institute for Cancer Research, Uppsala Branch). The PDGF-Rβ mutants used in the present study were: Y740/751F, Y763F, Y763/1009F, Y771F, Y775F, Y775/778F, Y934F, and Y1009/1021F (Table 1). Non-transfected PAE (PAE-NT) cells were used as a negative control. Dulbecco’s Modified Eagle Medium (DMEM) and Ham’s F12 media with Glutamax were used for culturing BJ fibroblasts and PAE cells, respectively (Gibco-BRL Life Technologies, Gaithersburg, MD). The media were supplemented with 10% fetal bovine serum (FBS) (Biowest, Nuaillé, France) and cells were cultured at 37 °C and 5% CO_2_.

### Reagents

Native bovine dermal collagen type I (3 mg/mL, 97% pure) was from Advanced Biomatrix (San Diego, CA). PDGF-BB was from Gibco-BRL Life Technologies (Stockholm, Sweden). The pan-cofilin antibody was from Abcam (Cambridge, UK). The pSer^3^-cofilin antibody (C-8992), tubulin antibody (T6074), LY294008, U73122, GÖ6976, bisindolylmaleimide-II, 4β-phorbol 12-myristate 13-acetate (PMA), wortmannin, and siRNAs were from Sigma-Aldrich. The PI3K p85 antibody (#4292) and the PLCγ1 (#5690) were from Cell Signaling. The siLentFect Lipid Reagent for siRNA transfection and Any KD Mini-PROTEAN TGX Precast protein gels were from Bio-Rad. The pY857 rabbit polyclonal PDGF-Rβ antibody was raised against a glutathione S-transferase fusion protein containing the C-terminal amino acid residues of the PDGF-Rβ.

### Collagen gel contraction

Collagen gel contraction was performed essentially as described previously^14^. In short, cells were trypsinized, suspended in serum-free DMEM and brought to the required cell density. The cell suspension was mixed on ice with collagen type I (CN-I) (5 vol 2 × DMEM, 1 vol 0.2 M HEPES pH 8.0, and 4 vol CN-I) at a 1:9 ratio. 100 μL of the mixture of cells and CN-I gel solution was added per well of a 96-well microtiter plate, previously blocked with 2% bovine serum (BSA) to avoid binding of the polymerizing gels to the wells. Plates were incubated at 37 °C for 1.5 h to allow collagen polymerization. Afterwards, the gels were floated with 100 μl serum-free DMEM in the absence or presence of PDGF-BB (20 ng/mL). Inhibitors were added at the indicated concentrations in the gels and in the floatation solutions. Collagen gel contraction was measured at the indicated time points and recorded as a decrease in gel area relative to the original gel area, using an inverted light microscope. The results are presented as the percentage of the original gel area after 2, 4, 8, and 24 h.

### Immunoblotting

Cells were seeded at a specific cell density in 6-well dishes and cultured overnight in complete medium. Afterwards cells were first pretreated with inhibitors for 20 min to 1 h in serum-free medium and then stimulated with PDGF-BB (20 ng/mL) for the indicated time points and subsequently washed and then lysed in RIPA buffer, supplemented with complete protease inhibitor tablets with EDTA from Roche (Basel, Switzerland) and 0.2 mM vanadate. Protein concentrations in supernatants were determined with the BCA kit (Thermo Fisher Scientific) and equal amounts of proteins were loaded onto Any kD Mini-PROTEAN TGX Precast Gels (Bio-Rad). After SDS-PAGE electrophoresis and transfer, membranes were first blocked in 5% BSA in TBS-tween and then probed with primary antibodies against pSer^3^-cofilin (1:4000), pan-cofilin (1:10000), tubulin (1:5000), PLCγ (1:1000), p85 (1:1000), and pY857 (1:1000) for 1.5 h at room temperature. Then, membranes were probed with HRP-conjugated donkey anti-rabbit antiserum as the secondary antibody (1:25000) for 1 h at room temperature. Protein bands were visualized using enhanced chemiluminescence (ECL) detection on a charge-coupled device (CCD) camera (Fuji, Minami-Ahigata, Japan). The protein bands were quantified using Aida Analyzer software. The quantified values of p-cofilin protein bands were subtracted by the quantified values of total-cofilin protein bands, and then they were normalized to the value of the control condition. The quantified data are presented as a bar-chart or written values underneath each corresponding band.

### Knock down of cofilin

Predesigned MISSION siRNA for human cofilin 1 (siRNA ID: SASI_Hs01_00078353), siRNA for porcine cofilin (sequences: NM_001004043-248_s: CCCUCUAUGACGCCACCUAdTdT and NM_001004043-248_as: UAGGUGGCGUCAUAGAGGGdTdT), and MISSION siRNA Universal Negative Control #1 were purchased from Sigma-Aldrich. For siRNA transfection, cells were first cultured in 6-well plates (75,000 cells per well) in full cell culture media (antibiotic-free) overnight. Then after 24 h, cells were transfected using siLentFect Lipid Reagent (from Bio-Rad) according to the manufacturer’s instructions at a final concentration of 100 nM siRNA for BJ fibroblast and 50 nM siRNA for PAE cells. Cells were harvested during 24–96 h and total cofilin levels were determined by immunoblotting. Transfected cells were used in collagen gel contraction assays.

## Electronic supplementary material

Supplementary information
