# Supplementary material for: PDGF-BB enhances collagen gel contraction through a PI3K-PLCγ-PKC-cofilin pathway
Source: Sci Rep. 2017 Aug 21;7:8924. doi: 10.1038/s41598-017-08411-1 (PMC5566449; doi:10.1038/s41598-017-08411-1)
Supplement: Supplementary file 1 — Supplementary information [file 41598_2017_8411_MOESM1_ESM.pdf]

PDGF-BB enhances collagen gel contraction through a PI3K-PLCγ-PKC-cofilin pathway

Vahid Reyhani, Maria Tsioumpekou, Tijs van Wieringen, Lars Rask, Johan Lennartsson, Kristofer Rubin

---

Supplementary figure 1:

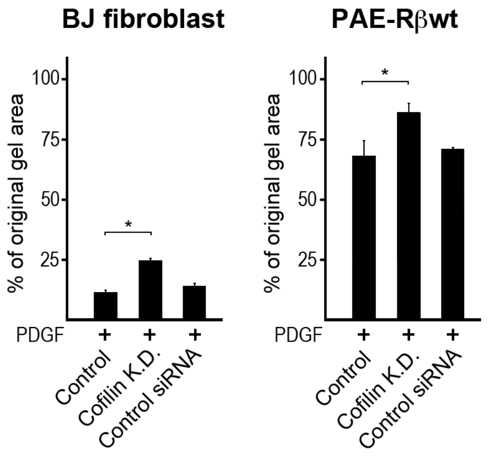

Fig. S1: Cofilin K.D. has similar effect on collagen gel contraction by both BJ-fibroblasts and PAE-Rβwt cells.
Comparison of the effect of cofilin knockdown in BJ fibroblasts and PAE-Rβwt cells 24h after initiation of contraction. In both panels the values are averaged from three individual experiments. Error bars are SEM and (*) refers to $p<0.05$.

Supplementary figure 2:

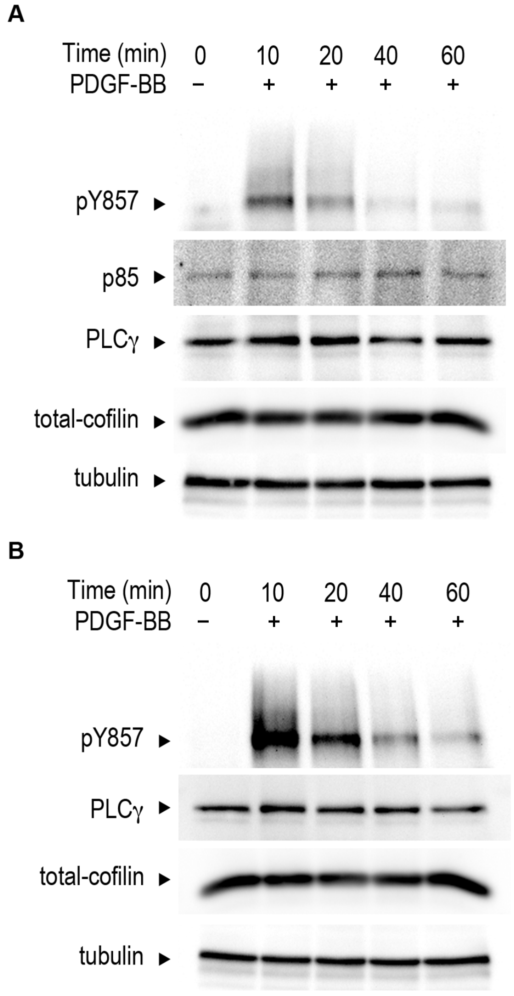

Fig. S2. Activation of PDGF-Rβ does not affect the total protein level of PI3K, PLCγ, cofilin, and tubulin.

(A) The total protein level of PI3K (p85), PLCγ, cofilin, and tubulin in BJ fibroblasts after activation and phosphorylation of PDGF-Rβ (pY857) upon stimulation with PDGF-BB (10, 20, 40, and 60 min). **(B)** The protein levels of PLCγ, cofilin, and tubulin in PAE-Rβ cells upon activation and phosphorylation of PDGF-Rβ (pY857) after 10, 20, 40, and 60 min stimulation with PDGF-BB. Due to technical difficulties with the antibody, the detection of PI3K in PAE-Rβ cells (unlike in BJ fibroblasts) was not successful.

The followings are the full-length blots that were used in figure 3A, figure 4, and figure 5.

Figure 3A

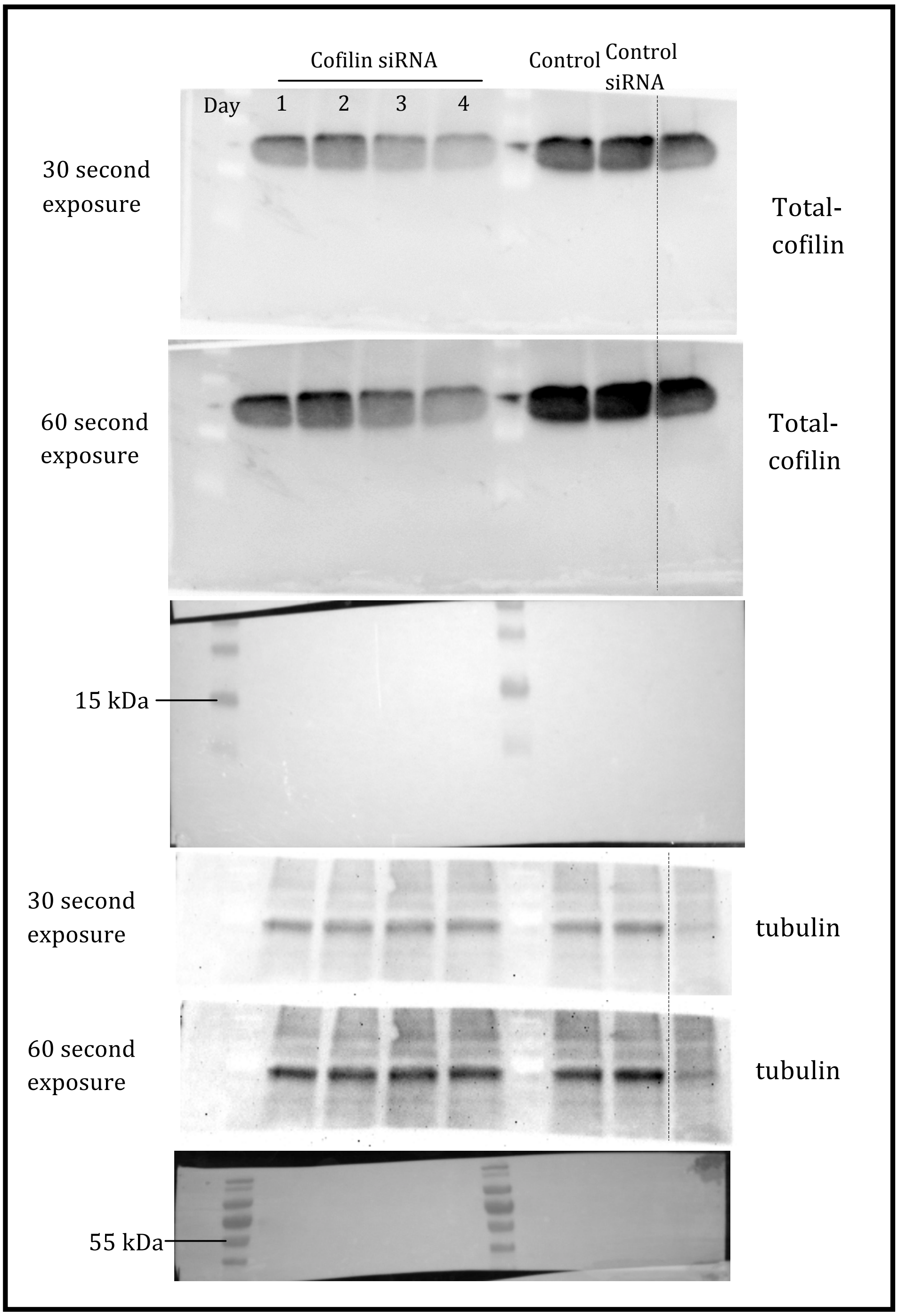

Last lane has irrelevant sample.

Figure 4

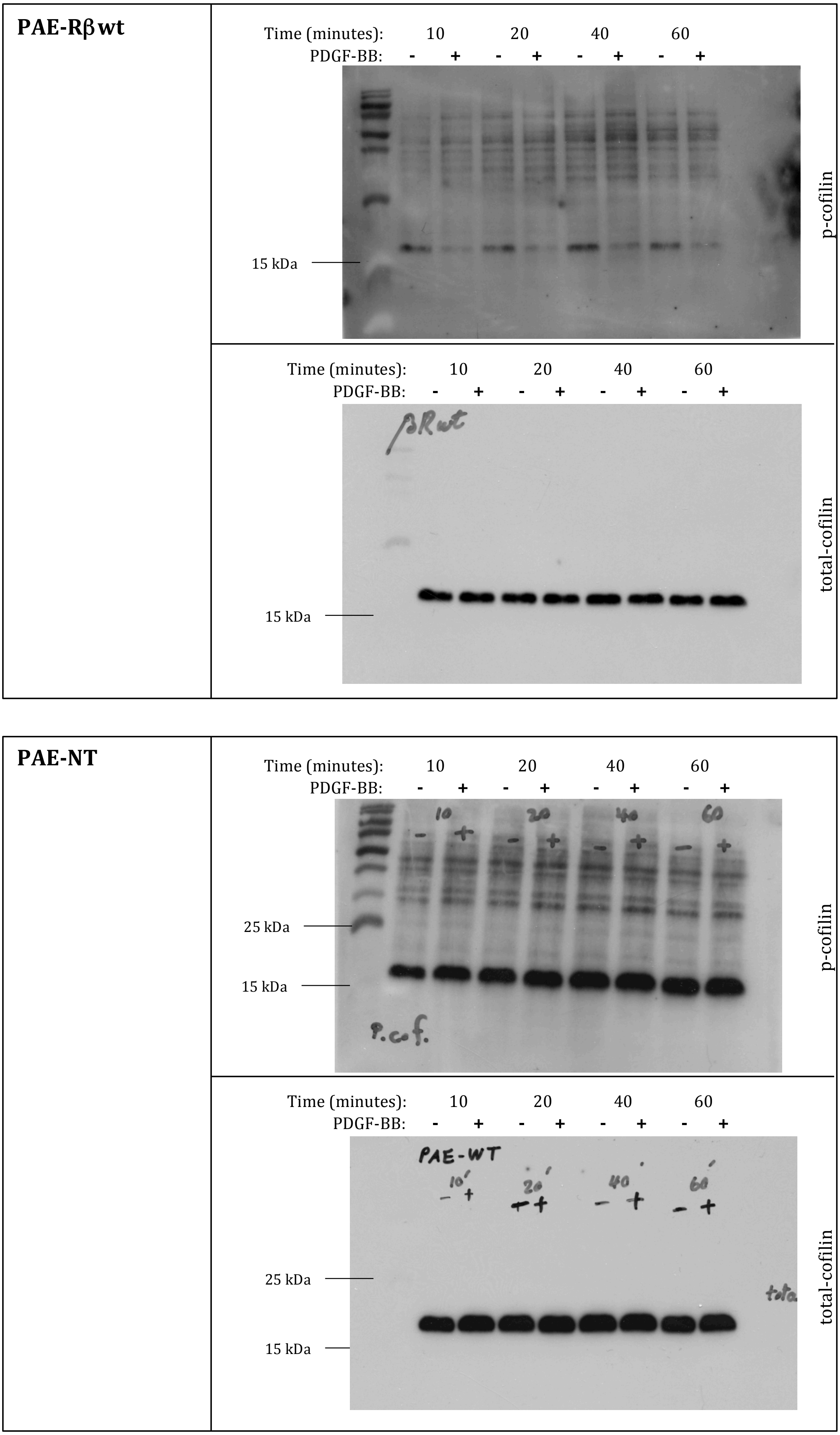

PAE-Rβwt
Time (minutes): 10 20 40 60
PDGF-BB: - + - + - + - +
15 kDa
p-cofilin
Time (minutes): 10 20 40 60
PDGF-BB: - + - + - + - +
15 kDa
total-cofilin
PAE-NT
Time (minutes): 10 20 40 60
PDGF-BB: - + - + - + - +
25 kDa
15 kDa
p-cofilin
Time (minutes): 10 20 40 60
PDGF-BB: - + - + - + - +
PAE-WT
25 kDa
15 kDa
total-cofilin

Figure 4

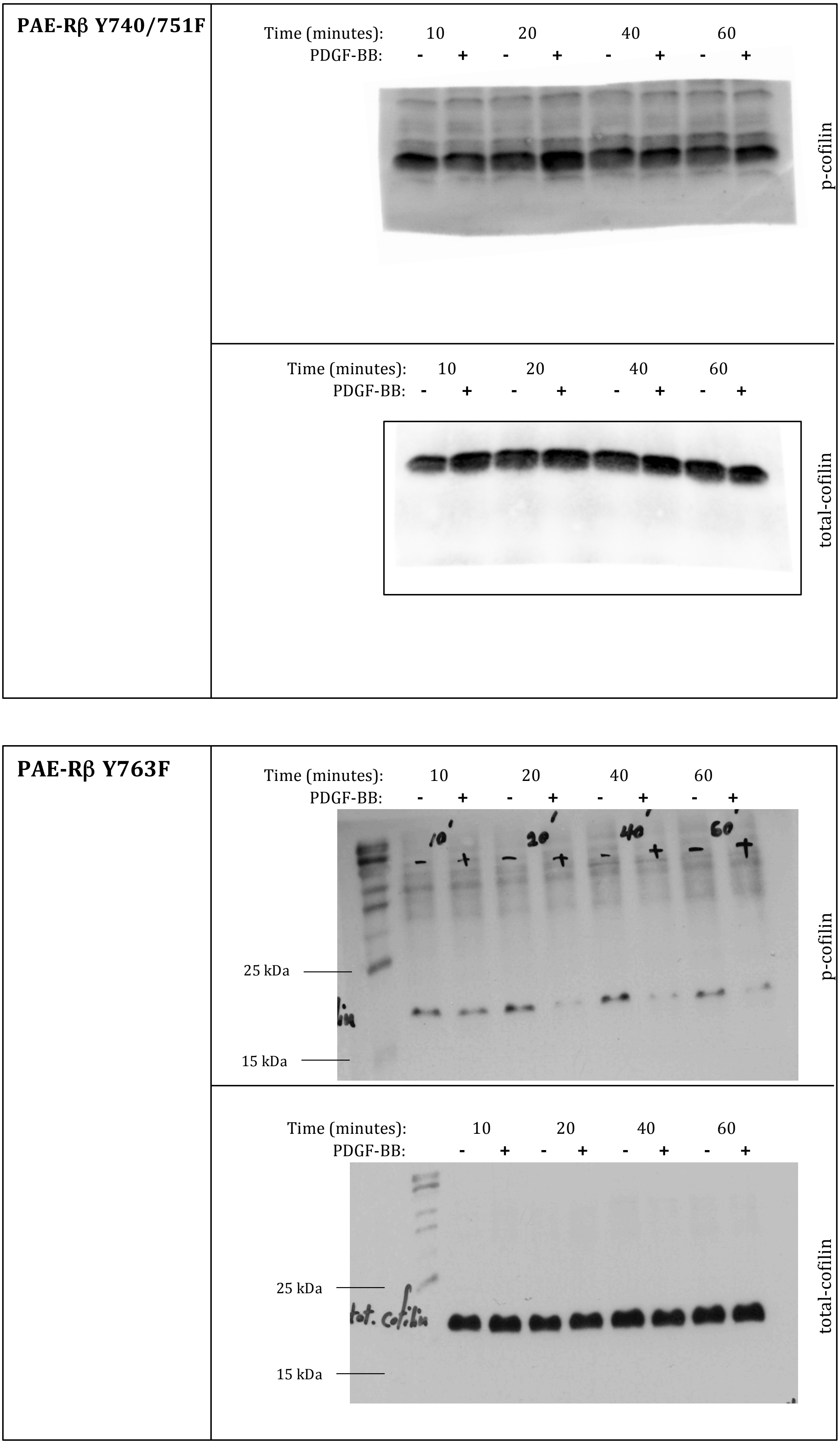
PAE-Rβ Y740/751F
Time (minutes): 10 20 40 60
PDGF-BB: - + - + - + - +
p-cofilin
Time (minutes): 10 20 40 60
PDGF-BB: - + - + - + - +
total-cofilin
PAE-Rβ Y763F
Time (minutes): 10 20 40 60
PDGF-BB: - + - + - + - +
25 kDa
15 kDa
p-cofilin
Time (minutes): 10 20 40 60
PDGF-BB: - + - + - + - +
25 kDa
tot. cofilin
15 kDa
total-cofilin

Figure 4

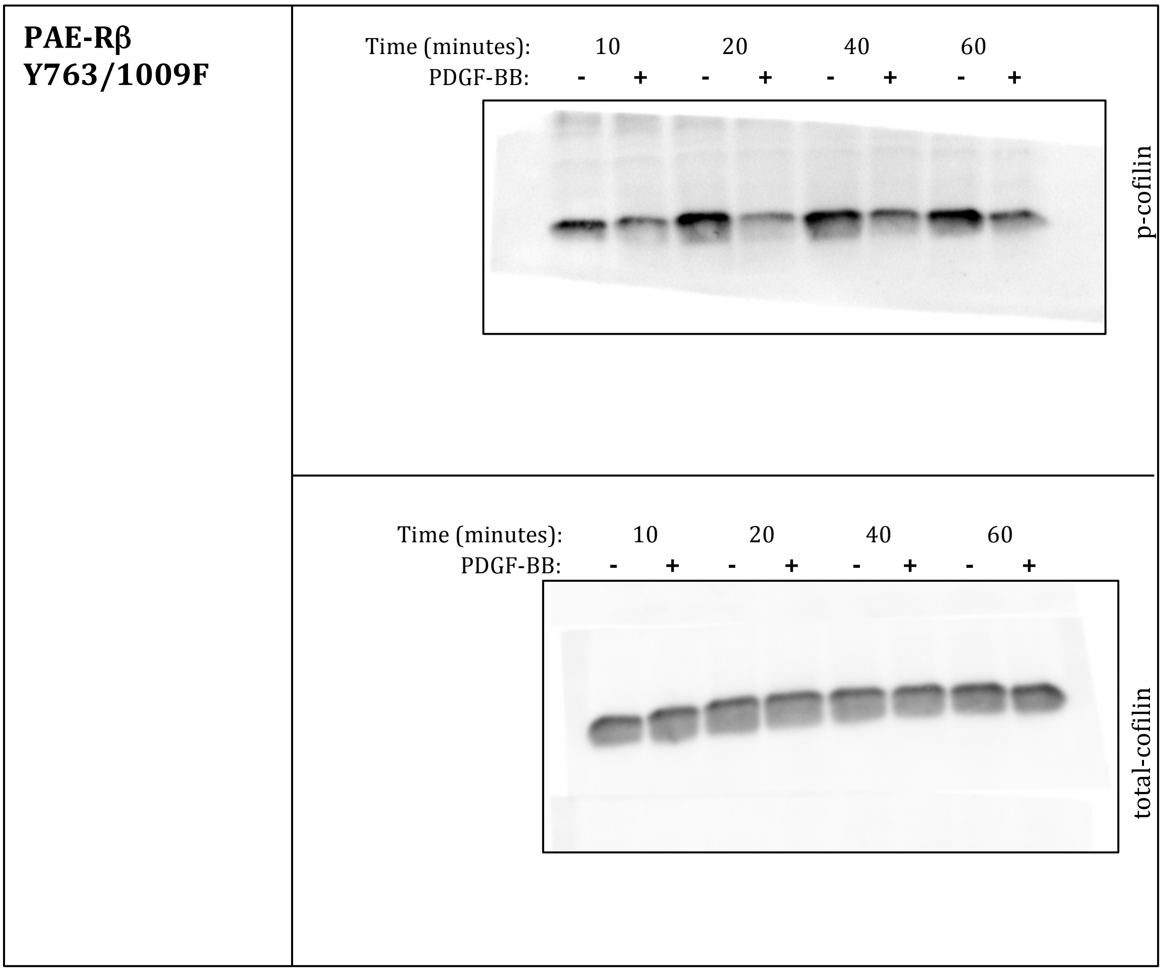

PAE-Rβ Y763/1009F
Time (minutes): 10 20 40 60
PDGF-BB: - + - + - + - +
p-cofilin
Time (minutes): 10 20 40 60
PDGF-BB: - + - + - + - +
total-cofilin

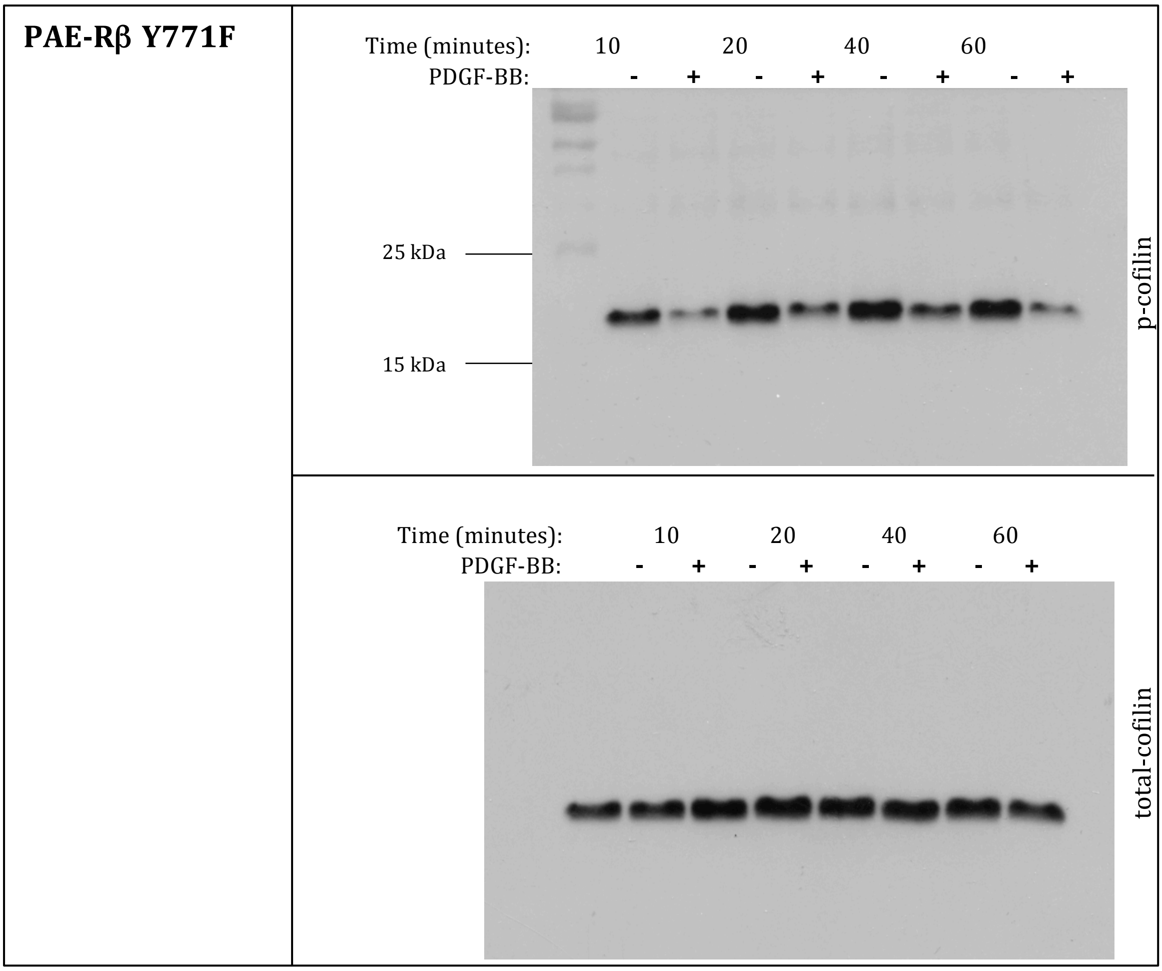

PAE-Rβ Y771F
Time (minutes): 10 20 40 60
PDGF-BB: - + - + - + - +
25 kDa
15 kDa
p-cofilin
Time (minutes): 10 20 40 60
PDGF-BB: - + - + - + - +
total-cofilin

Figure 4

PAE-Rβ Y775F

Time (minutes): 10 20 40 60

PDGF-BB: - + - + - + - +

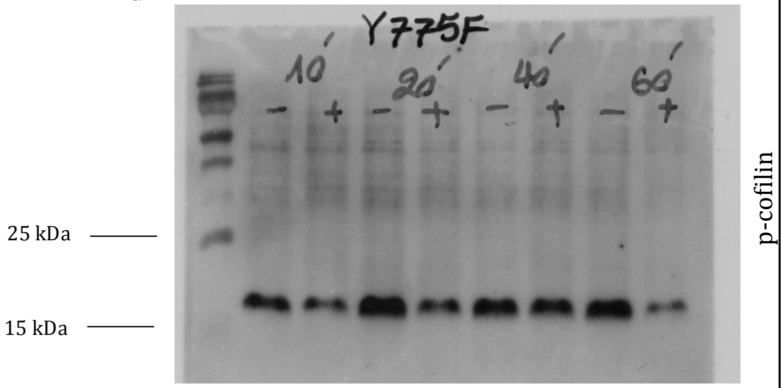

Time (minutes): 10 20 40 60

PDGF-BB: - + - + - + - +

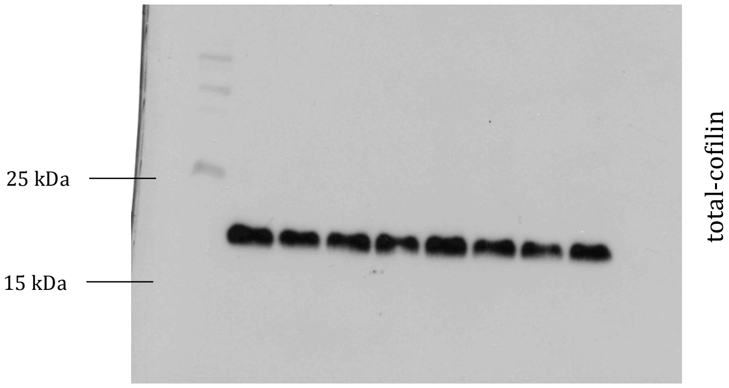

PAE-Rβ Y775/778F

Time (minutes): 10 20 40 60

PDGF-BB: - + - + - + - +

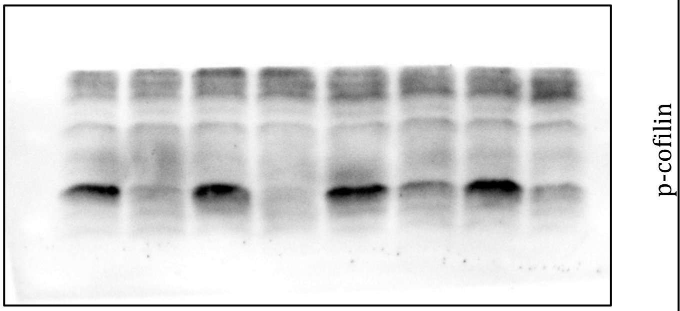

Time (minutes): 10 20 40 60

PDGF-BB: - + - + - + - +

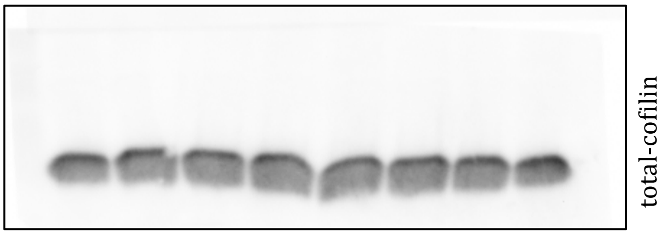

Figure 4

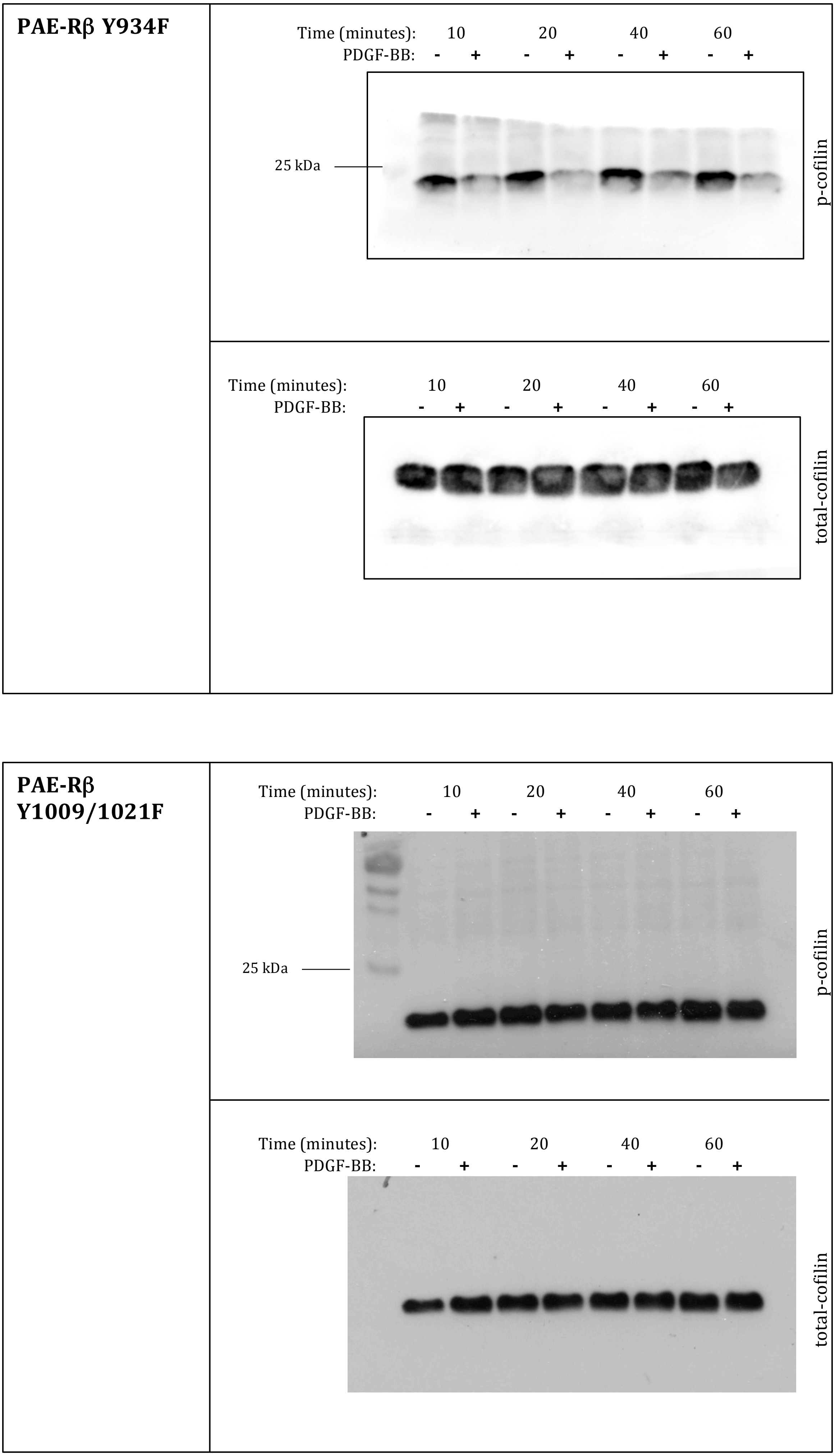
PAE-Rβ Y934F
Time (minutes): 10 20 40 60
PDGF-BB: - + - + - + - +
25 kDa
p-cofilin
Time (minutes): 10 20 40 60
PDGF-BB: - + - + - + - +
total-cofilin
PAE-Rβ Y1009/1021F
Time (minutes): 10 20 40 60
PDGF-BB: - + - + - + - +
25 kDa
p-cofilin
Time (minutes): 10 20 40 60
PDGF-BB: - + - + - + - +
total-cofilin

Figure 5A

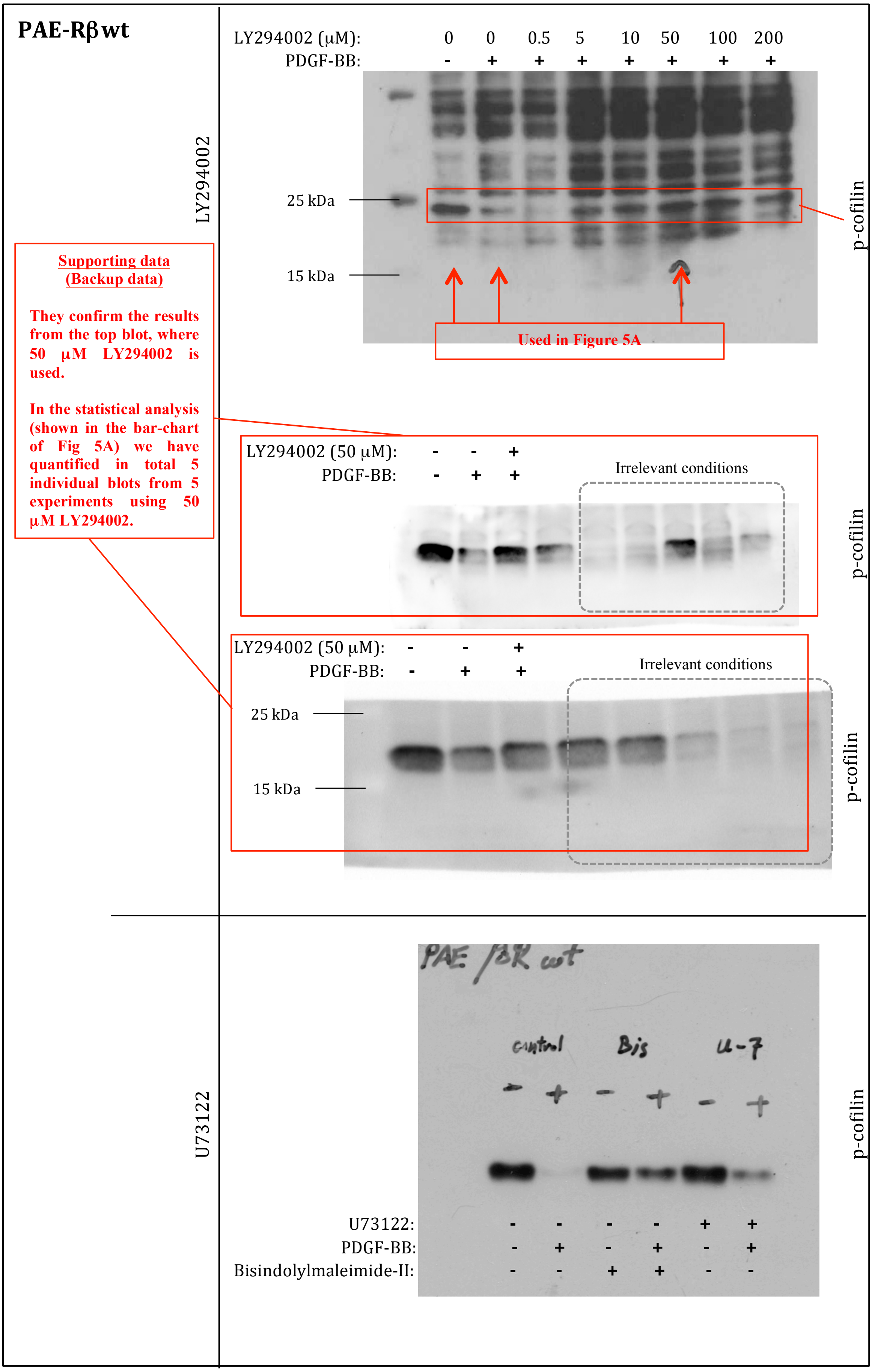
PAE-Rβwt
LY294002
LY294002 (μM): 0 0 0.5 5 10 50 100 200
PDGF-BB: - + + + + + + +
25 kDa
15 kDa
p-cofilin
Used in Figure 5A
Supporting data (Backup data)
They confirm the results from the top blot, where 50 μM LY294002 is used.
In the statistical analysis (shown in the bar-chart of Fig 5A) we have quantified in total 5 individual blots from 5 experiments using 50 μM LY294002.
LY294002 (50 μM): - - +
PDGF-BB: - + +
Irrelevant conditions
p-cofilin
LY294002 (50 μM): - - +
PDGF-BB: - + +
Irrelevant conditions
25 kDa
15 kDa
p-cofilin
U73122
PAE /βR wt
control
Bis
p-cofilin
U73122: - - - - + +
PDGF-BB: - + - + - +
Bisindolylmaleimide-II: - - + + - -

Figure 5B

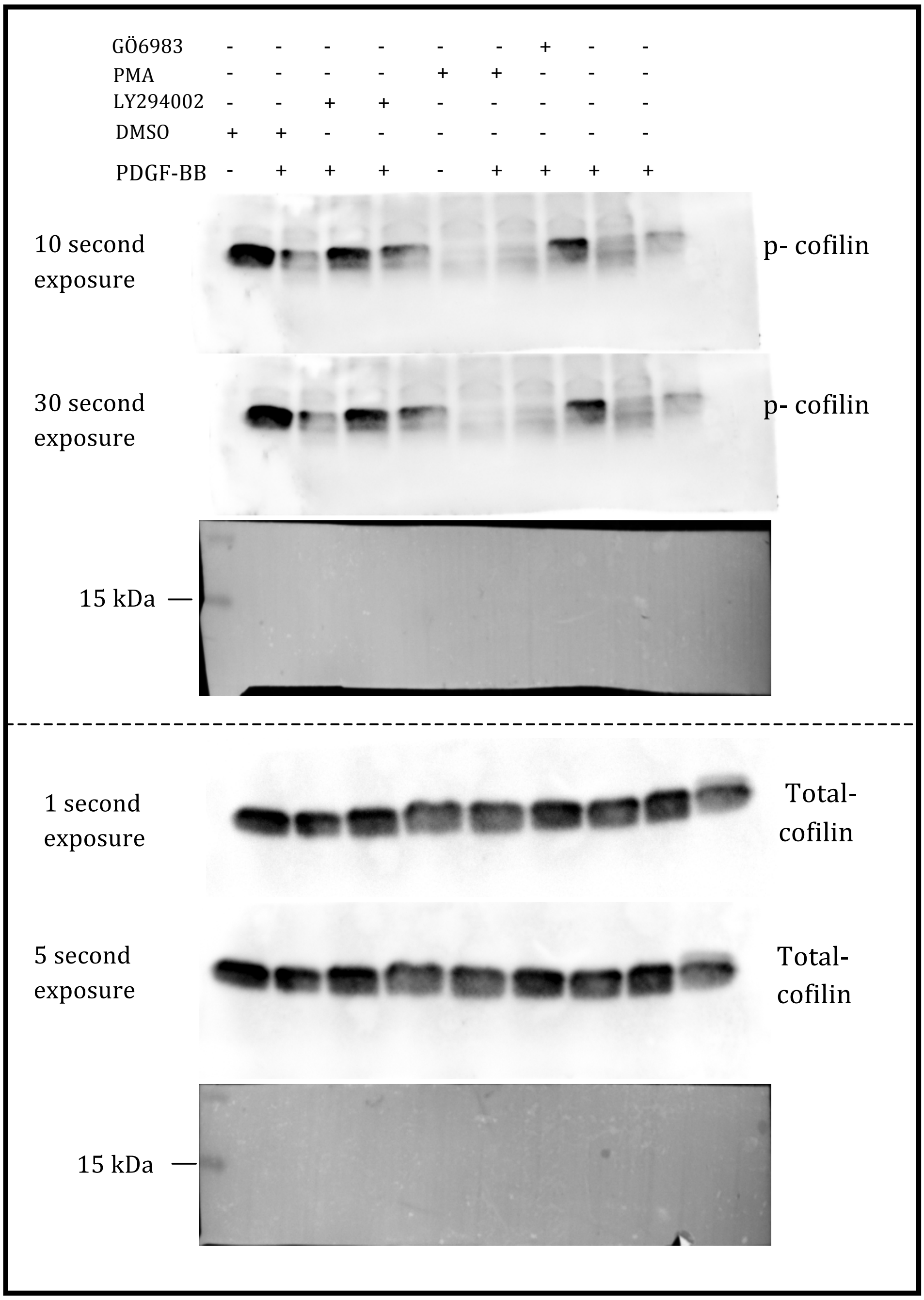

GÖ6983 - - - - - - + - -
PMA - - - - + + - - -
LY294002 - - + + - - - - -
DMSO + + - - - - - - -
PDGF-BB - + + + - + + + +
10 second exposure
p- cofilin
30 second exposure
p- cofilin
15 kDa
1 second exposure
Total-cofilin
5 second exposure
Total-cofilin
15 kDa

Figure 5C

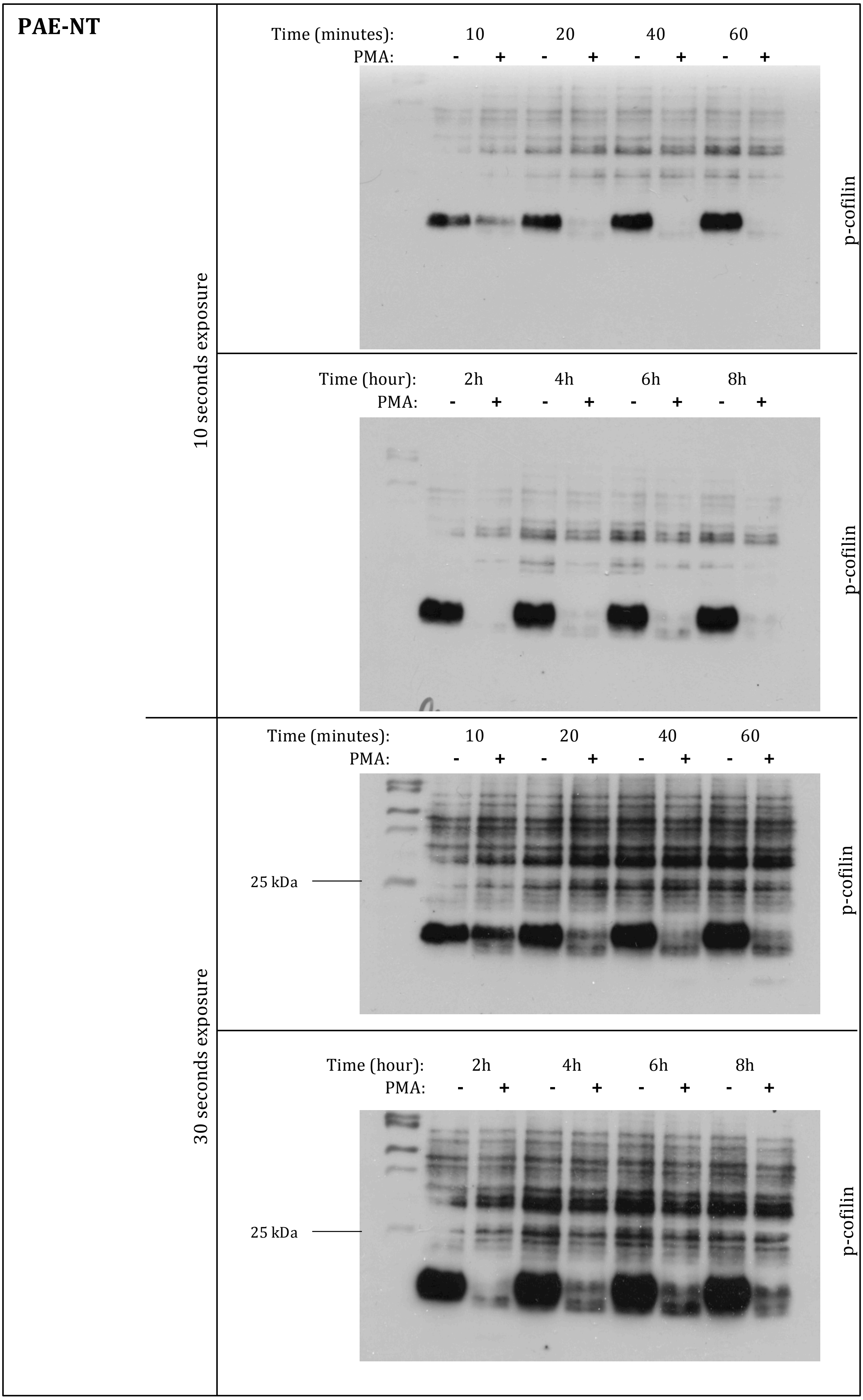

PAE-NT
10 seconds exposure
30 seconds exposure
Time (minutes): 10 20 40 60
PMA: - + - + - + - +
Time (hour): 2h 4h 6h 8h
PMA: - + - + - + - +
25 kDa
p-cofilin

Figure 5C

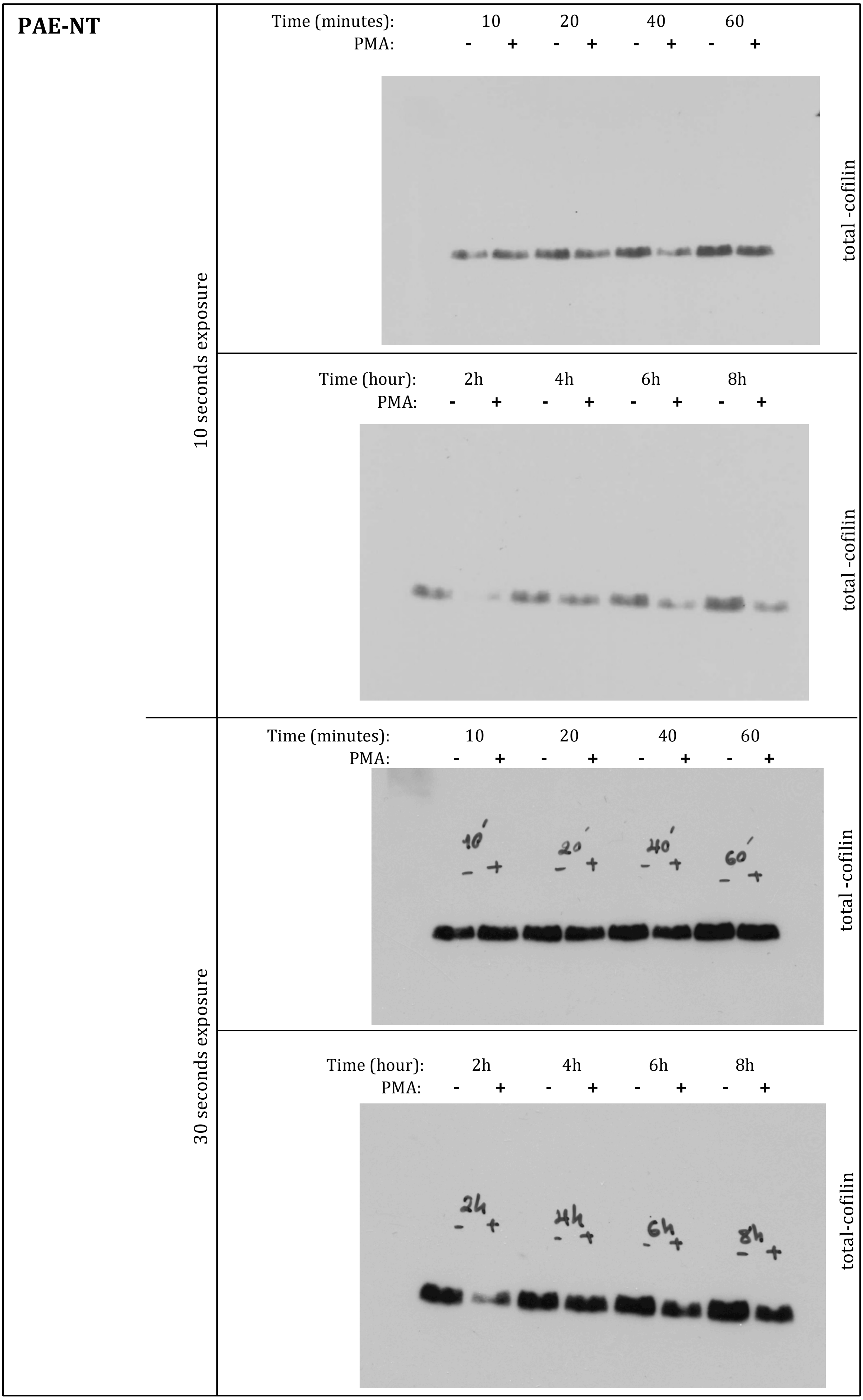
PAE-NT
10 seconds exposure
Time (minutes): 10 20 40 60
PMA: - + - + - + - +
total -cofilin
Time (hour): 2h 4h 6h 8h
PMA: - + - + - + - +
total -cofilin
30 seconds exposure
Time (minutes): 10 20 40 60
PMA: - + - + - + - +
total -cofilin
Time (hour): 2h 4h 6h 8h
PMA: - + - + - + - +
total-cofilin

Figure 5D

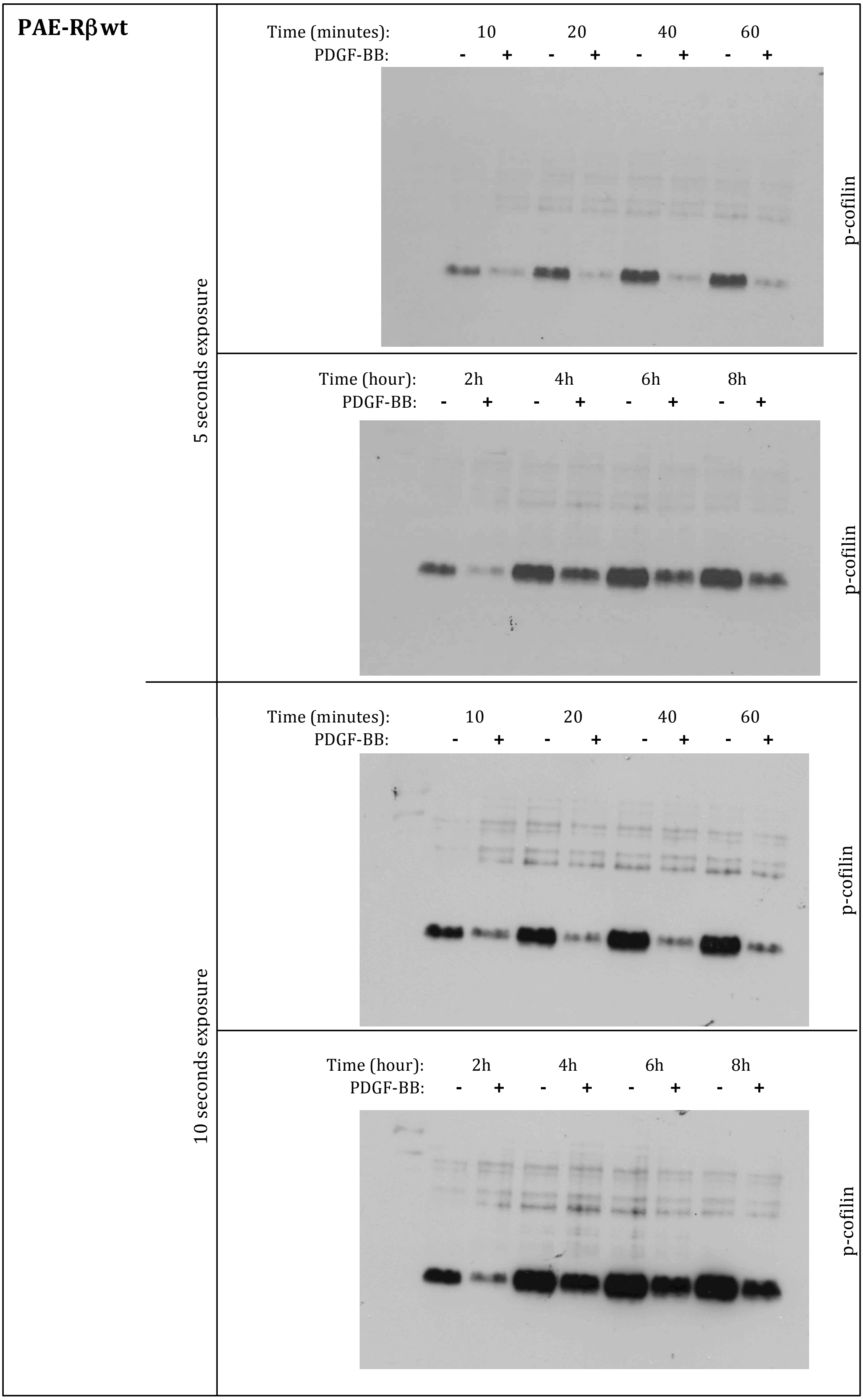
PAE-Rβwt
5 seconds exposure
10 seconds exposure
Time (minutes): 10 20 40 60
PDGF-BB: - + - + - + - +
Time (hour): 2h 4h 6h 8h
PDGF-BB: - + - + - + - +
p-cofilin

Figure 5D

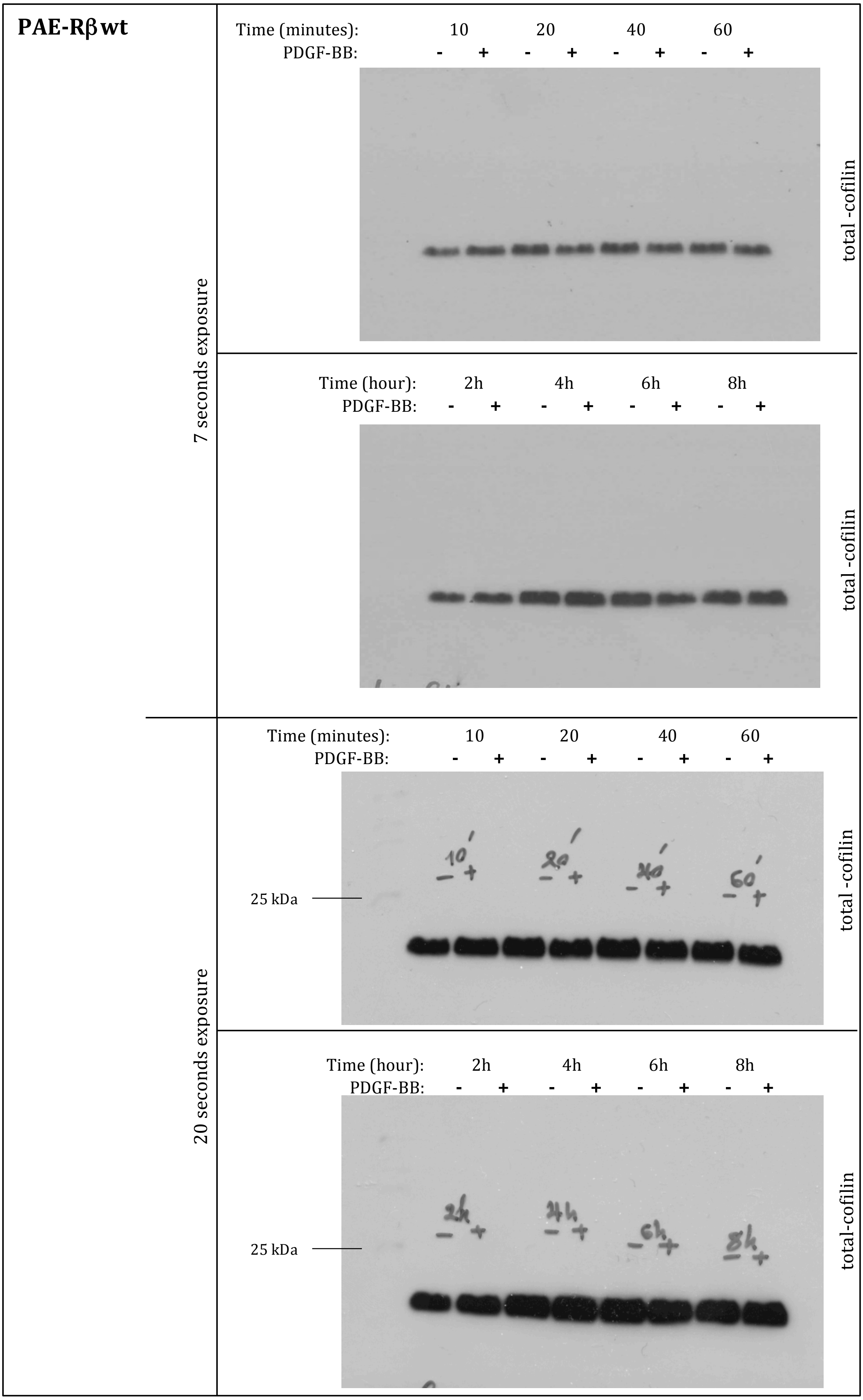
PAE-Rβwt
7 seconds exposure
Time (minutes): 10 20 40 60
PDGF-BB: - + - + - + - +
total -cofilin
Time (hour): 2h 4h 6h 8h
PDGF-BB: - + - + - + - +
total -cofilin
20 seconds exposure
Time (minutes): 10 20 40 60
PDGF-BB: - + - + - + - +
25 kDa
total -cofilin
Time (hour): 2h 4h 6h 8h
PDGF-BB: - + - + - + - +
25 kDa
total-cofilin
